# Supplementary material for: Assigning the Absolute Configuration of Inositol Poly- and Pyrophosphates by NMR Using a Single Chiral Solvating Agent
Source: Biomolecules. 2023 Jul 19;13(7):1150. doi: 10.3390/biom13071150 (PMC10377360; doi:10.3390/biom13071150)

Supplementary Materials

Assigning the Absolute Configuration of Inositol Poly- and Pyrophosphates by NMR Using a Single Chiral Solvating Agent

Kevin Ritter, Nikolaus Jork, Anne-Sophie Unmüßig, Maja Köhn and Henning J. Jessen*

Table of Contents

1. General Remarks
2. Syntheses Adapted From Literature
3. Synthesis of *rac*-1,4,5-$InsP_3$ (**14**)
4. Synthesis of *rac*-1,4,5,6-$InsP_4$ (**18**)
5. Synthesis of 4/6-PCP-$InsP_5$ (**23**)
6. Synthesis of 5-PP-1/3-$InsP_1$ (**28**)
7. Literature
8. NMR Spectra

1. General Remarks

Reactions were carried out using flame-dried glassware under an atmosphere of dry Argon and magnetically stirred, unless noted otherwise. Air- and moisture-sensitive liquids and solutions were transferred via syringe or stainless steel canula.

Reagents were purchased from commercial suppliers (Acros, Sigma-Aldrich, Fluka, TCI) and used without further purification, unless noted otherwise.

Solvents were obtained in analytical grade and used as received for extractions, precipitation and solid washing.

Dry solvents for reactions were purchased in a dry form from Sigma-Aldrich and stored over molecular sieves as well as under the atmosphere of dry N_2.

Deuterated solvents for NMR and reactions were obtained from Armar Chemicals, Switzerland and euriso-top, Germany, in the indicated purity grade and used as received for NMR spectroscopy.

Strong ion-exchange chromatography was performed using an automated Äkta® – system. Q-Sepharose was purchased from Sigma-Aldrich. Buffer solutions were produced manually using milliQ H_2O.

Preparative RP-MPLC was performed using PuriFlash 5.125 by Interchim. The AQsolid phase was purchased from Interchim.

Lyophilizations were done with Christ Freeze Dryer Alpha 1-4 LDplus and Christ Freeze Dryer Alpha 1-2 LDplus.

^{1}H-NMR spectra were recorded on Bruker 300 MHz spectrometers, Bruker 400 MHz (with cryoprobe) and Bruker 500 MHz spectrometers in the indicated deuterated solvent. Data are reported as follows: chemical shift (δ, ppm), multiplicity (s, singlet; d, doublet; t, triplet; q, quartet; m, multiplet; br. s, broad signal), coupling constant(s) (J, Hz), integration. All signals were referenced to the internal solvent signal as standard (D_2O, δ 4.79; MeCN-d_3, δ 1.94, $CDCl_3$, 7.26).

^{13}C{^{1}H}-NMR spectra were recorded with ^{1}H-decoupling on Bruker 126 MHz, Bruker 101 MHz (with cryoprobe) spectrometers at 298K in the indicated deuterated solvent.

^{31}P{^{1}H}-NMR spectra and **^{31}P-NMR** spectra were recorded with ^{1}H-decoupling or ^{1}H coupling, respectively, on Bruker 202 MHz, 162 MHz (with cryoprobe) and Bruker 122

MHz spectrometers in the indicated deuterated solvent. All signals were referenced to an internal standard (PPP).

Mass spectra were recorded by C. Warth (Mass spectrometry service of the University of Freiburg) on a Thermo LCQ Advantage [spray voltage: 2.5 – 4.0 kV, spray current: 5 µA, ion transfer tube: 250 (150) °C, evaporation temperature: 50 – 400°C.

2. Syntheses Adapted From Literature

2.1 Synthesis of Phosphoramidites

Synthesis of AB-Phosphoramidite **S1**

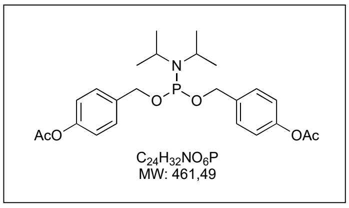

The compound was synthesized according to *Jessen et al*. Analytical data are in accordance with literature[1].

Synthesis of PCP-Phosphoramidite **S2**

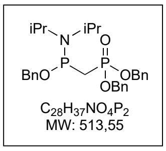

The compound was synthesized according to *Hostachy et al*. Analytical data are in accordance with literature[2].

Synthesis of XE-Phosphoramidite **S3**

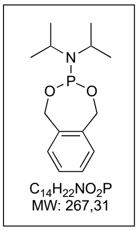

The compound was synthesized according to *Zhan et al.* Analytical data are in accordance with literature [3].

Synthesis of Bn-Phosphoramidite **S4**

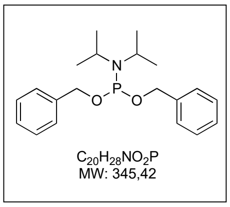

The compound was synthesized according to *Das et al*. Analytical data are in accordance with literature [4].

2.2 Synthesis of PP-InsP5 and PP2-InsP4 Compounds

Synthesis of 1-PP-InsP5 (**3**) & 3-PP-InsP5 (**7**)

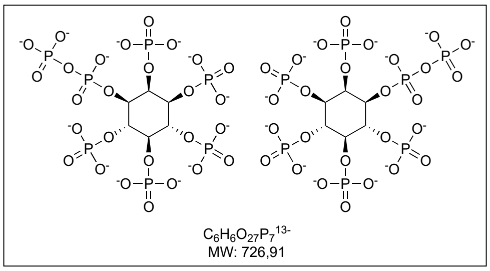

The compound was synthesized according to *Capolicchio et al*. Analytical data are in accordance with literature[5].

Synthesis of 4-PP-InsP5 (**6**) & 6-PP-InsP5 (**4**)

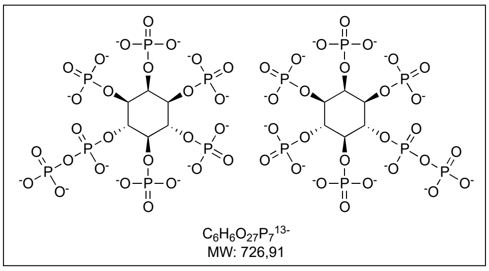

The compound was synthesized according to *Capolicchio et al*. Analytical data are in accordance with literature [5].

Synthesis of 5-PP-InsP5 (**5**)

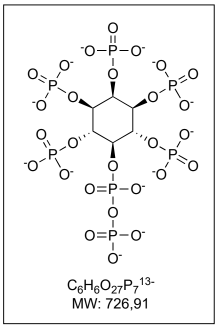

The compound was synthesized according to *Haas et al.* Analytical data are in accordance with literature [6].

Synthesis of 1,5-PP2-InsP4 (**S5**) & 3,5-PP2-InsP4 (**S6**)

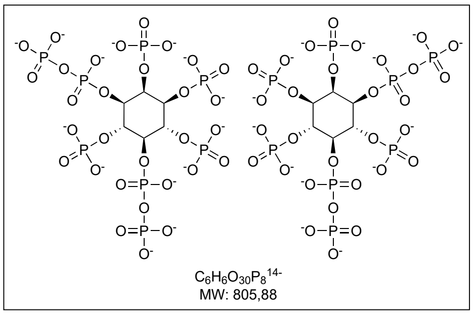

The compound was synthesized according to *Capolicchio et al.* Analytical data are in accordance with literature [7].

3. Synthesis of *rac*-1,4,5-InsP3 (**14**)

3.1 Synthesis of Protected Inositol Derivative **9**

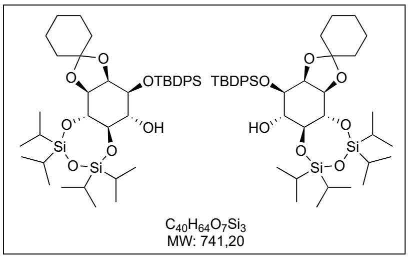

The compound was synthesized according to the literature. Analytical data are in accordance with literature [8–10].

3.2 Synthesis of Protected Inositol Derivative **11**

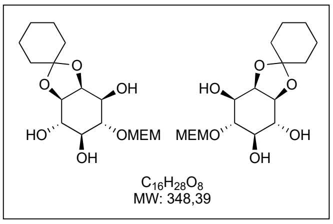

Starting material **9** (200 mg, 270 µmol, 1.0 eq) was dissolved in $CHCl_3$ (2 ml), in a pressure tube. MEM-Cl (335 mg, 307 µL, 270 µmol, 10.0 eq.) and DIPEA (530 mg, 716 µL, 405 µmol, 15.0 eq.) were added. The reaction mixture was heated to 70°C for 3 days. The reaction mixture was diluted with CH:EE (1:1, 10 mL), after reaching room temperature and filtered over silica in a fritted funnel. The silica was rinsed with CH:EE (1:1, 200 mL) and solvent was removed under reduced pressure to obtain the protected inositol **10**.

10 was dissolved in TBAF (1 mL, 1M in THF) and heated to 70°C for 3 days in a pressure tube. The reaction was diluted with DCM (20 mL) and water (20 mL) after reaching room temperature. The aqueous layer was extracted with CH_2Cl_2 (3 × 40 mL). The combined aqueous layers were dried over $MgSO_4$ and concentrated under reduced pressure. The crude product was purified by silica column chromatography (cyclohexane/EtOAc = 1:1 → pure EtOAc). The target compound was obtained as colorless solid (94 mg, 270 mmol, quant.).

^{1}H-NMR (400 MHz, $CDCl_3$): δ = 4.92 (d, *J* = 1.7 Hz, 2H), 4.42 (dd, *J* = 5.3, 4.1 Hz, 1H), 4.02 (dd, *J* = 7.6, 5.3 Hz, 1H), 3.84 – 3.76 (m, 3H), 3.75 – 3.65 (m, 2H), 3.59 – 3.54 (m, 2H), 3.38 (s, 3H), 3.31 (dd, *J* = 10.1, 8.7 Hz, 1H), 1.78 – 1.52 (m, 8H), 1.39 (dd, *J* = 7.8, 4.6 Hz, 2H) ppm.

^{13}C-NMR (101 MHz, $CDCl_3$): δ = 110.8, 97.1, 83.1, 78.0, 75.4, 75.2, 72.8, 71.6, 70.1, 67.8, 59.0, 37.8, 25.0, 23.9, 23.5 ppm.

HRMS (ESI) $[M+H]^+$ calculated for $C_{16}H_{29}O_8$: 349.1857, found 349.1857.

3.3 Synthesis of Protected $InsP_3$ Derivative **12**

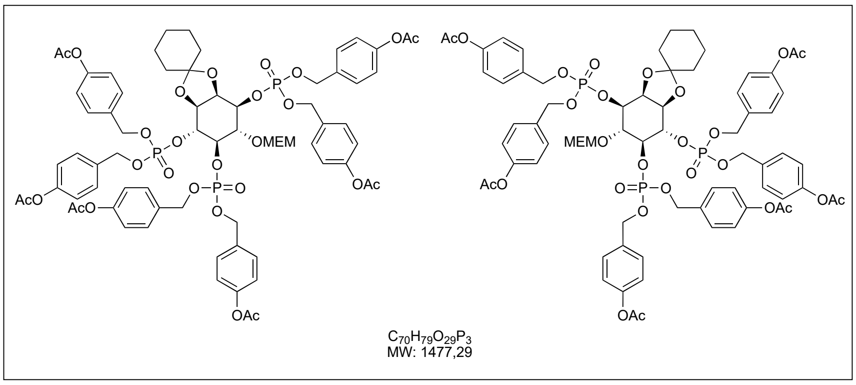

Starting material **11** (25 mg, 72 µmol, 1.0 eq.) and AB-phosphoramidite **S1** (165 mg, 359 µmol, 4.0 eq.) were coevaporated with MeCN (2 mg) and then dissolved in MeCN (5 mL). A solution of ETT (1M in MeCN, 349 µL, 349 µmol, 4.0 eq.) was added and reaction mixture was stirred for 30 min at room temperature. Oxidation was achieved by the addition of *m*CPBA (70%, 89 mg, 359 µmol, 4.0 eq.) at 0°C. Solvent was removed under reduced pressure and the crude product was purified by automated reversed phase MPLC (Interchim C18-aq-Column, H_2O/MeCN, gradient: 35 - 100% MeCN). The target compound was obtained as a colorless solid (60 mg, 41 µmol, 56%).

^{1}H-NMR (400 MHz, $CDCl_3$): δ = 7.46 – 7.18 (m, 12H), 7.15 – 6.95 (m, 12H), 5.15 – 4.94 (m, 12H), 4.94 – 4.70 (m, 4H), 4.67 (dd, *J* = 6.5, 3.8 Hz, 1H), 4.52 (td, *J* = 8.7, 5.7 Hz, 1H), 4.34 (dd, *J* = 7.8, 5.7 Hz, 1H), 4.29 (t, *J* = 6.7 Hz, 1H), 3.69 (ddt, *J* = 26.4, 11.0, 4.6 Hz, 2H), 3.41 (t, *J* = 4.6 Hz, 2H), 3.29 (s, 3H), 2.32 – 2.29 (m, 18H), 1.84 – 1.16 (m, 10H) ppm.

^{13}C-NMR (101 MHz, $CDCl_3$): δ = 169.24, 169.22, 169.20, 169.18, 169.16, 150.80, 150.78, 150.71, 150.67, 150.64, 133.58, 133.51, 133.41, 133.33, 133.26, 133.20, 133.18, 133.13, 129.28, 129.24, 129.17, 129.15, 129.11, 129.04, 121.79, 121.75, 121.68, 121.64, 111.68, 96.24, 79.19 – 78.68 (m), 75.62 – 75.49 (m), 75.40, 73.19 – 73.04 (m), 71.66, 69.09, 69.03, 68.97, 68.92, 68.86, 68.85, 68.79, 67.85, 58.87, 36.54, 34.29, 29.70, 24.86, 23.89, 23.59, 21.11 ppm.

^{31}P{^{1}H}-NMR (162 MHz, $CDCl_3$): δ = -1.64, -1.98, -2.23 ppm.

HRMS (ESI) $[M+Na]^+$ calculated for $C_{70}H_{79}O_{29}NaP_3$: 1499.3812, found 1499.3798.

3.4 Synthesis of *rac*-1,4,5-InsP3 (**14**)

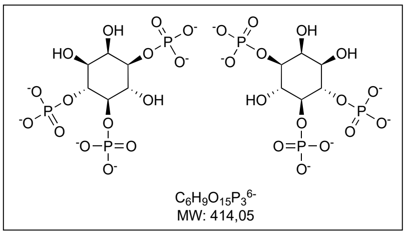

Starting material **12** (30 mg, 20 µmol, 1.0 eq) was dissolved in a solution of piperidine in DMF (1 mL, 20%, v/v) and stirred for 1.5 h at room temperature. A precipitate was formed by the addition of Et_2O (13 mL) and the supernatant was separated after centrifugation. The precipitate was washed with Et_2O (13 mL) and dried under HV to obtain intermediate **13**.
The intermediate **13** was used without further purification. It was dissolved in a solution of HCl in ethanol (1.25M, 1 mL) and stirred for 3 days at room temperature. All volatiles were removed under reduced pressure. The residual was dissolved in water (0.5 mL) and acetone (6 mL). NaI was added, until no more precipitate was formed. Supernatant was separated after centrifugation and the precipitate was washed with acetone (6 mL) and dried under HV. The product was obtained as a colorless solid (5.7 mg, 12 µmol, 60%).

^{1}H-NMR (400 MHz, D_2O): δ = 4.30 (q, *J* = 9.2 Hz, 1H), 4.24 (t, *J* = 2.8 Hz, 1H), 4.12 – 3.96 (m, 2H), 3.88 (t, *J* = 9.5 Hz, 1H), 3.70 (dd, *J* = 9.8, 2.8 Hz, 1H) ppm.
^{31}P{^{1}H}-NMR (162 MHz, D_2O): δ = 0.80, 0.30, -0.30 ppm.
^{13}C-NMR (101 MHz, D_2O): δ = 78.46, 77.19, 75.17, 70.69 (2 × C), 70.04 ppm.
HRMS (ESI) $[M]^-$ calculated for $C_6H_{14}O_{15}P_3$: 418.9551, found 418.9566.

4. Synthesis of *rac*-1,4,5,6-InsP4 (**18**)

4.1 Protected Inositol Derivative **15**

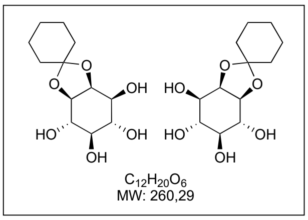

The compound was synthesized according to *Kiely et al*. Analytical data are in accordance with literature[8].

4.2 Synthesis of InsP4 derivative **16**

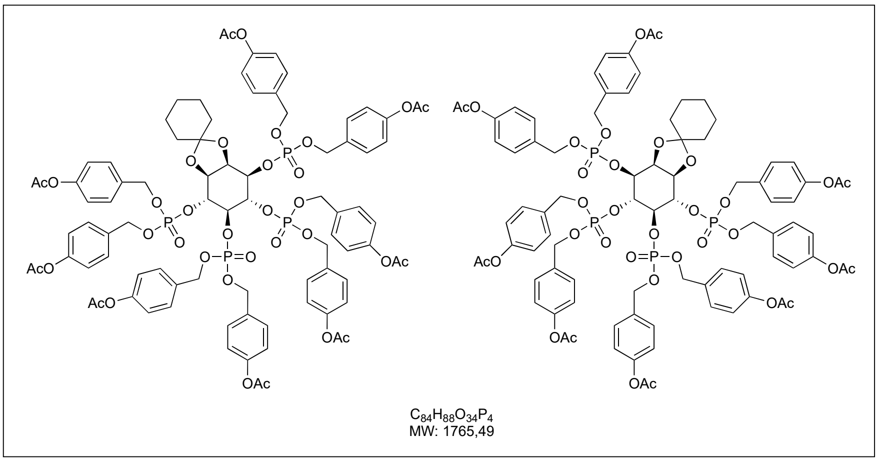

Inositol derivative **15** (60.0 mg, 231 μmol, 1.0 eq) was dissolved in DMF (5 ml) and AB-P-amidite (**S1**) (798 mg, 1.73 mmol, 7.5 eq.) and DCI (204 mg, 1.73 mmol, 7.5 eq) was added. The reaction was stirred for 45 min at room temperature. The reaction progress was followed by ^{31}P-NMR. The mixture was cooled to 0°C and *m*CPBA (70%, 426 mg, 1.73 mmol, 7.5 eq.) was added and the reaction was stirred for 15 min at room temperature. The reaction progress was followed by ^{31}P-NMR. The solvent was removed under reduced pressure and crude product was purified by automated reversed phase MPLC (Interchim C18-HP-Column, H_2O/MeCN, gradient: 20 - 100% MeCN) to obtain the title compound **16** (243 mg, 138 μmol, 60%) as a colorless oil.

^{1}**H-NMR** (400 MHz, $CDCl_3$): δ = 7.37 – 6.88 (m, 32H), 5.15 – 4.87 (m, 18H), 4.82 – 4.66 (m, 3H), 4.31 (dd, *J* = 6.6, 5.6 Hz, 1H), 2.33 – 2.25 (m, 24H), 1.78 – 1.24 (m, 10H) ppm.

^{31}**P{^{1}H}-NMR** (162 MHz, $CDCl_3$): δ = -1.64, -2.06, -2.22, -2.28 ppm.

^{13}**C-NMR** (101 MHz, $CDCl_3$): δ = 169.95 – 168.68 (m), 151.49 – 149.64 (m), 133.97 – 132.22 (m), 129.91 – 128.53 (m), 122.54 – 120.87 (m), 111.88, 78.46, 75.13, 73.33, 72.83, 69.27 – 68.79 (m), 36.05, 33.80, 24.87, 23.90, 23.55, 21.11 ppm.

HRMS (ESI) $[M+Na]^+$ calculated for $C_{84}H_{88}O_{34}NaP_4$: 1787.4000, found 1787.4023.

4.3 Synthesis of $InsP_4$ derivative **17**

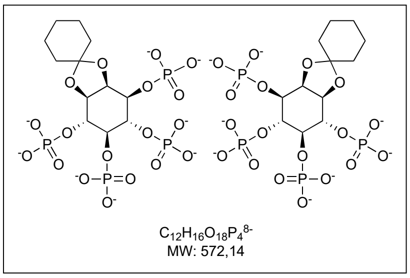

$InsP_4$ derivative **16** (80.0 mg, 45.3 μmol, 1.0 eq.) was dissolved in a mixture of piperidine (2 ml) and DMF (4 ml). The mixture was stirred for 2.5 h at room temperature. The reaction progress was followed by ^{31}P-NMR. The crude product was obtained by precipitation with a solution of $NaClO_4$ in acetone (1M, 40 ml). After centrifugation, the supernatant was discarded and the precipitate was washed with acetone. After another centrifugation, the precipitate was dissolved in water (0.5 mL) and was precipitated with a solution of $NaClO_4$ in acetone (1M, 40 mL). After centrifugation, the supernatant was discarded and the precipitate was washed with acetone to obtain the title compound **17** (28 mg, 42 mmol, 93%) as a colorless solid.

^{1}**H-NMR** (400 MHz, D_2O): δ = 4.86 (dd, *J* = 7.7, 3.0 Hz, 1H), 4.79 – 4.73 (m, 1H), 4.50 – 4.25 (m, 4H), 1.87 – 1.64 (m, 3H), 1.64 – 1.42 (m, 4H), 1.42 – 1.22 (m, 3H) ppm.

^{31}**P{^{1}H}-NMR** (162 MHz, D_2O): δ = 4.42, 3.20, 2.81, 2.48 ppm.

^{13}**C-NMR** (101 MHz, D_2O): δ = 110.68, 81.15, 76.11, 74.42, 74.33, 71.48, 70.39, 34.15, 32.31, 24.46, 23.53, 23.12 ppm.

HRMS (ESI) $[M-H]^-$ calculated for $C_{12}H_{24}O_{18}P_4$: 578.9840, found 578.9843.

4.4 Synthesis of *rac*-1,4,5,6-$InsP_4$ (**18**)

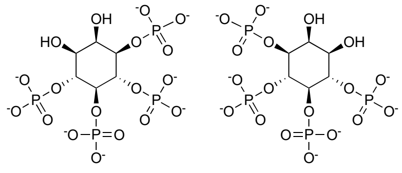

$InsP_4$ derivative **17** (25.0 mg, 43.7 µmol, 1.0 eq.) was dissolved in H_2O (2 mL) and trifluoroacetic acid (50 µL, 74.0 mg, 64.9 µmol, 14.9 eq) was added. The reaction was stirred for 1h at 60°C. The reaction progress was followed by ^{31}P-NMR. The reaction was stopped by adding NEt_3 (90 µL, 65.7 mg, 64.9 µmol, 14.9 eq.). The crude product was obtained by precipitation with a solution of $NaClO_4$ in acetone (1M, 40 ml). After centrifugation, the supernatant was discarded and the precipitate was washed with acetone. The title compound **18** (17 mg, 35 µmol, 79%) was obtained as colorless solid.

^{1}H NMR (400 MHz, D_2O): δ = 4.50 (q, *J* = 9.4 Hz, 1H), 4.38 (q, *J* = 9.3 Hz, 1H), 4.29 – 4.17 (m, 3H), 3.75 (dd, *J* = 9.8, 2.8 Hz, 1H) ppm.

^{31}P{^{1}H}-NMR (162 MHz, D_2O): δ = 0.46, 0.18, 0.14, -0.46 ppm.

^{13}C-NMR (101 MHz, D_2O): δ = 77.48 (2 × C), 76.49, 74.71, 70.70, 69.48 ppm.

HRMS (ESI) $[M-H]^-$ calculated for $C_6H_{15}O_{18}P_4$: 498.9214, found 498.9213.

5. Synthesis of 4/6-PCP-InsP5 (23)

5.1 Synthesis of Protected Diol **19**

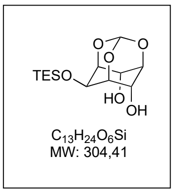

The compound was synthesized according to *Capolicchio et al.* Analytical data are in accordance with literature [5].

5.2 Synthesis of PCP-Derivative **20**

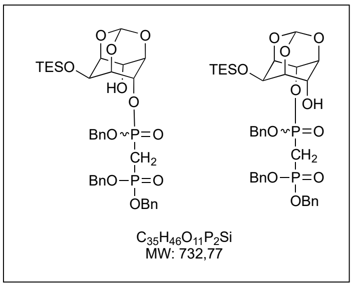

Diol **19** (1.33 g, 4.36 mmol, 2.8 eq.) and PCP-phosphoramidite **S2** (800 mg, 1.56 mmol, 1.0 eq.) were coevaporated (2 × 3 mL) with dry MeCN and dissolved in dry MeCN (4 mL). A solution of ETT in MeCN (1 M, 1.71 mL, 221 mg, 1.71 mmol, 1.1 eq.) was added and the mixture was stirred for 1 h at r.t. The reaction progress was followed by ^{31}P-NMR. The mixture was cooled to 0°C and ^{t}BuOOH (5.50 M in decane, 566 µL, 281 mg, 3.12 mmol, 2.0 eq.) was added. The reaction was stirred for 30 min at r.t. Half of the solvent was removed under reduced pressure and EtOAc (150 mL) was added. The organic layer was washed with H_2O (2 × 100 mL) and brine (100 mL) and dried over Na_2SO_4. The solvent was removed under reduced pressure. The crude product was purified by silica column chromatography (cyclohexane/EtOAc = 3:1 → pure EtOAc) to obtain the title compound **20** (400 mg, 546 mmol, 35%) as a colorless solid.

The compound was obtained as a 2:1 mixture of two diastereomers:

¹H-NMR (400 MHz, $CDCl_3$): δ = 7.33 – 7.13 (m, 45H), 5.44 (d, *J* = 1.3 Hz, 2H), 5.42 (d, *J* = 1.3 Hz, 1H), 5.19 (dtd, *J* = 10.6, 3.9, 1.7 Hz, 2H), 5.11 – 4.87 (m, 14H), 4.86 – 4.81 (m, 6H), 4.52 (td, *J* = 3.8, 1.8 Hz, 2H), 4.42 (td, *J* = 3.7, 1.8 Hz, 1H), 4.39 (tt, *J* = 3.5, 1.6 Hz, 2H), 4.32 (q, *J* = 1.7 Hz, 1H), 4.25 (q, *J* = 1.8 Hz, 2H), 4.17 (dq, *J* = 3.9, 1.9 Hz, 1H), 4.10 – 4.05 (m, 5H), 3.80 (dq, *J* = 3.9, 1.9 Hz, 2H), 2.44 – 2.21 (m, 6H), 0.94 – 0.86 (m, 27H), 0.67 – 0.50 (m, 18H) ppm.

³¹P{¹H}-NMR (162 MHz, $CDCl_3$) δ = 20.13 (4P), 19.89 (d, *J* = 10.0 Hz, 1P), 18.89 (d, *J* = 10.0 Hz, 1P).

HRMS (ESI) $[M+H]^+$ calc. for $C_{35}H_{47}O_{11}P_2Si$: 733.2318, found 733.2363.

5.3 Synthesis of Protected PCP-InsP₅ derivative **22**

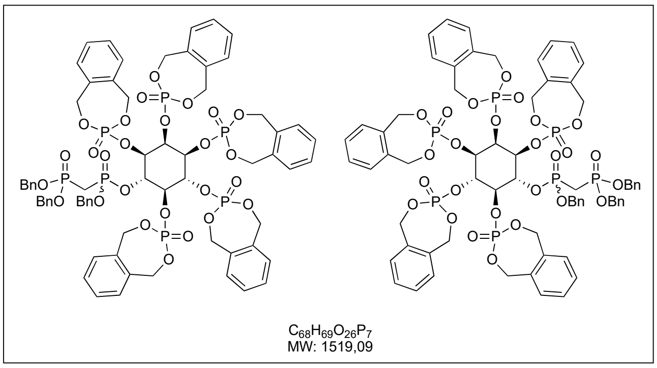

PCP-derivative **20** (100 mg, 136 mmol, 1.0 eq.) was dissolved in a solution of *p*TsOH in MeOH (2 mM, 8 mL) and the solution was stirred for 3h at 60°C. The reaction progress was followed by ³¹P-NMR. The reaction was quenched by adding a solution of NEt_3 in MeOH (2 mM, 8 mL) and the solvent was removed under reduced pressure. The crude product was dissolved in dichloromethane (8 mL) and AB-P-amidite (**S1**) (1.01 g, 2.18 mmol, 16 eq.) and ETT (282 mg, 2.18 mmol, 16 eq) were added. The reaction was stirred for 2 h at room temperature. The reaction progress was followed by ³¹P-NMR. The mixture was cooled to 0°C and *m*CPBA (70%, 538 mg, 2.18 mmol, 16 eq.) was added and the reaction was stirred for 15 min at room temperature. The reaction progress was followed by ³¹P-NMR. The solvent was removed under reduced pressure and crude product was purified by automated reversed phase MPLC (Interchim C18-HP-Column, H_2O/MeCN, gradient: 20 - 100% MeCN) to obtain the title compound **22** (65 mg, 43.0 µmol, 31%) as a colorless solid (mixture of two diastereomers).

The compound was obtained as a 2:1 mixture of two diastereomers:

^{1}H-NMR (400 MHz, $CDCl_3$): δ = 7.52 – 6.72 (m, 35H), 5.87 – 4.60 (m, 32H), 3.21 – 2.69 (m, 2H) ppm.

^{31}P{^{1}H}-NMR (162 MHz, $CDCl_3$) δ = 21.34, 20.33, 20.29, 20.20, 20.18, -1.36, -1.77, -2.05, -2.51, -2.53, -2.56, -3.11, -3.23, -4.29, -4.48 ppm.

HRMS (ESI) $[M+Na]^+$ calc. for $C_{68}H_{69}O_{26}NaP_7$: 1541.2133, found 1541.2134.

5.4 Synthesis of 4,6-PCP-InsP$_5$ (**23**)

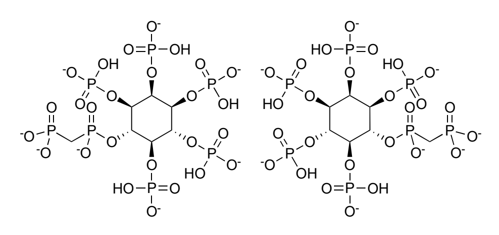

PCP-InsP$_5$ derivative **22** (60.0 mg, 39.5 µmol, 1.0 eq.) was dissolved in tBuOH/H_2O (10 mL, 40:7). $NaHCO_3$ (43.0 mg, 516 µmol, 13.1 eq.) and Pd/C (10% on activated charcoal, 120.0 mg) were added and the reaction was hydrogenated 2h at 40 bar (autoclave). The catalyst was removed by filtration and the aqueous phase was extracted with CH_2Cl_2 (2 × 25 mL) and freeze-dried. The crude product purification by SAX-column chromatography (Q-Sepharose, HiTrap CaptoQ ImpRes, eluted by NH_4HCO_3-buffer Gradient, 1M). Lyophilization yielded the title compound **23** (33. mg, 28.3 µmol, 72%) as a colorless solid.

^{1}H-NMR (400 MHz, D_2O): δ = 4.82 – 4.74 (m, 1H), 4.43 (q, *J* = 9.8 Hz, 1H), 4.29 (q, *J* = 9.7 Hz, 1H), 4.00 (t, *J* = 9.5 Hz, 1H), 3.96 – 3.88 (m, 2H), 2.19 (t, *J* = 20.1 Hz, 2H) ppm.

^{31}P{^{1}H}-NMR (162 MHz, D_2O): δ = 19.92 (d, *J* = 9.6 Hz, 1P), 14.42 (d, *J* = 9.4 Hz, 1P), 2.13 (1P), 1.90 (1P), 1.58 (1P), 1.50 (1P), 0.61 ppm.

^{13}C-NMR (101 MHz, D_2O) δ 76.65, 75.58, 74.61 (2 × C), 73.30, 72.93, 31.27 – 27.07 (m) ppm.

HRMS (ESI) $[M-H]^-$ calc. for $C_7H_{21}O_{26}P_7$: 736.8412, found 736.8370.

6. Synthesis of 5-PP-1/3-InsP1 (**28**)

6.1 Synthesis of (Fm)2 InsP1-derivative **24**

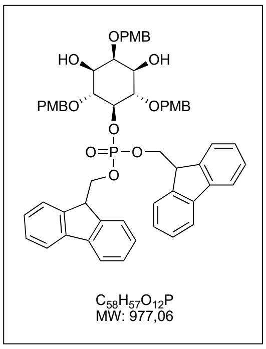

The compound was synthesized according to *Capolicchio et al.* Analytical data are in accordance with literature [7].

6.2 Synthesis of InsP2-derivative **25**

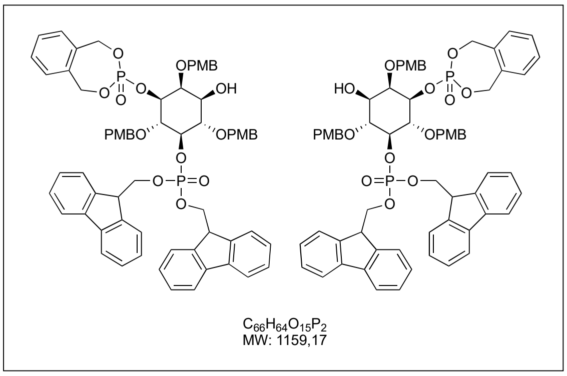

Diol **24** (300 mg, 307 µmol, 2.5 eq.) and XE-phosphoramidite (**S3**) (33.0 mg, 123 µmol, 1.0 eq.) were coevaporated (3 × 2 mL) with dry MeCN and dissolved in dry MeCN/CH_2Cl_2 (4 mL, 3:1). A solution of ETT in MeCN (1 M, 135 µL, 18.0 mg, 135 µmol 1.1 eq.) was added and the mixture was stirred at r.t. The reaction progress was followed by ^{31}P-NMR. After completion of the coupling (60 min), the mixture was cooled to 0°C and ^{t}BuOOH (5.50 M in decane, 44.6 µL, 22.1 mg, 246 µmol, 2.0 eq.) was added. The reaction was stirred for 30 min at r.t. Half of the solvent was removed under reduced pressure and EtOAc (40 mL) was added. The organic layer was washed with H_2O (2 × 20 mL) and brine (20 mL) and dried over $MgSO_4$. The solvent

was removed under reduced pressure. The crude product was purified by automated reversed phase MPLC (Interchim C18-HP-Column, H_2O/MeCN, gradient: 60 - 100% MeCN), followed by prep-HPLC (ProntoSIL C18-AQ-Column, H_2O/MeCN, gradient: 20 - 80% MeCN) and freeze-dried to obtain the title compound **25** (72.2 mg, 58.3 µmol, 48%) as a colorless solid.

^{1}H-NMR (300 MHz, MeCN-*d*$_3$): δ = 7.74 – 7.66 (m, 4H), 7.42 – 7.14 (m, 20H), 7.14 – 7.08 (m, 2H), 6.97 – 6.89 (m, 2H), 6.81 – 6.72 (m, 2H), 6.62 – 6.52 (m, 2H), 5.21 – 4.91 (m, 4H), 4.89 – 4.54 (m, 6H), 4.49 – 4.43 (m, 1H), 4.37 – 4.29 (m, 1H), 4.24 – 4.18 (m, 2H), 4.13 – 4.06 (m, 4H), 3.92 – 3.88 (m, 1H), 3.86 – 3.81 (m, 1H), 3.79 (s, 3H), 3.71 (s, 3H), 3.68 – 3.62 (m, 2H), 3.59 (s, 3H) ppm.
^{31}P{^{1}H}-NMR (122 MHz, MeCN-*d*$_3$): δ = - 2.54 (1P), - 3.51 (1P) ppm.
^{13}C-NMR (101 MHz, MeCN-*d*$_3$): δ = 130.39, 130.32 (d, *J* = 3.1 Hz), 130.13 (d, *J* = 5.1 Hz), 128.02 (d, *J* = 3.5 Hz), 127.98, 126.68, 126.03, 120.91 (dd, *J* = 5.4, 2.1 Hz), 114.62, 114.36, 114.24, 81.49 (dd, *J* = 7.2, 2.1 Hz), 78.27 (d, *J* = 5.2 Hz), 75.93, 75.13, 74.12, 72.34, 69.68, 69.60 (d, *J* = 4.3 Hz), 69.55 (d, *J* = 3.4 Hz), 69.39 (d, *J* = 7.2 Hz), 55.90, 55.81, 55.67, 48.59 (dd, *J* = 8.4, 3.1 Hz) ppm.
HRMS (ESI) [M+NH_4^+] calc. for $C_{66}H_{68}O_{15}NP_2$: 1176.4064, found 1176.4070.

6.3 Synthesis of $InsP_2$-derivative **26**

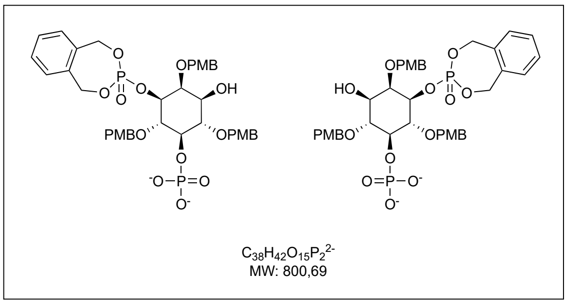

Intermediate **25** (35.0 mg, 30.0 µmol, 1.0 eq.) was dissolved in MeCN (660 µL) and piperidine (330 µL) was added. The mixture was stirred at r.t. for 3 d. The reaction progress was followed by ^{31}P-NMR. After completion of the deprotection the solvent was removed under reduced pressure and the crude product was purified by automated reversed phase MPLC (Interchim C18-HP-Column, H_2O/MeCN/TEAA buffer (100 mM), gradient: 10 - 95% MeCN + const. 5% buffer) and freeze-dried to obtain the title compound **26** (17.5 mg, 21.9 µmol, 72%) as a colorless solid.

^{1}H-NMR (300 MHz, MeOH-*d$_4$*): δ = 7.57 – 7.53 (m, 2H), 7.47 – 7.43 (m, 2H), 7.40 – 7.31 (m, 6H), 6.90 – 6.84 (m 4H), 6.79 – 6.74 (m, 2H), 5.06 (d, J = 9.8 Hz, 2H), 4.96 – 4.89 (m, 4H), 4.81 – 4.68 (m, 4H), 4.25 (t, J = 2.4 Hz, 1H), 4.19 – 4.11 (m, 1H), 4.04 – 4.00 (m, 2H), 3.98 – 3.94 (m, 1H), 3.80 (s, 3H), 3.78 (s, 3H), 3.75 – 3.73 (m, 1H), 3.67 (s, 3H) ppm.

^{31}P{^{1}H}-NMR (162 MHz, MeOH-*d$_4$*): δ = 0.14 (1P), - 0.37 (1P) ppm.

^{13}C-NMR (101 MHz, MeCN-*d$_3$*): δ = 132.27, 131.50, 131.26, 130.80, 130.70, 130.28, 129.83, 129.03, 128.36, 113.07, 80.62, 79.47, 74.83, 74.60, 73.95, 71.47, 57.51, 54.27, 54.23, 54.18 ppm.

HRMS (ESI) $[M+C_5 H_{12}N^+]^{2+}$ calc. for $C_{43}H_{56}O_{15}NP_2$: 888.2974, found 888.3120.

6.4 Synthesis of PP-InsP$_1$-derivative **27**

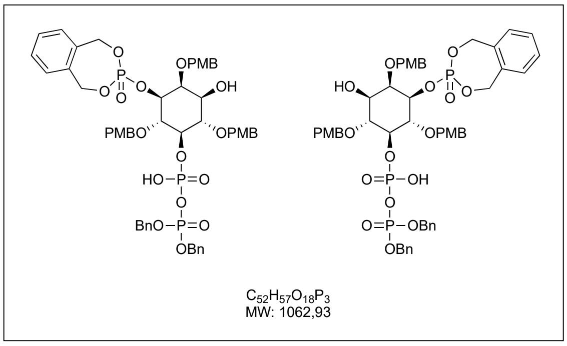

InsP$_2$ derivative **26** (15.0 mg, 19.0 µmol, 1.0 eq.) and Bn-phosphoramidite (**S4**) (7.82 mg, 21.0 µmol, 1.1 eq.) were coevaporated (3 × 2 mL) with dry MeCN and dissolved in dry MeCN/CH_2Cl_2 (3 mL, 2:1). The mixture was cooled to 0°C and a solution of ETT in MeCN (1 M, 60.0 µL, 8.00 µg, 60.0 µmol, 4.0 eq.) was added dropwise over 30 min and the mixture was stirred 60 min at 0°C and 45 min at r.t. The reaction progress was followed by ^{31}P-NMR. The mixture was cooled to 0°C and ^{t}BuOOH (5.50 M in decane, 6.82 µL, 3.38 µg, 37.5 µmol, 2.0 eq.) was added. The reaction was stirred for 30 min at r.t. Half of the solvent was removed under reduced pressure and EtOAc (40 mL) was added. The organic layer was washed with H_2O (2 × 20 mL) and brine (20 mL) and dried over $MgSO_4$. The solvent was removed under reduced pressure. The crude product was purified by automated reversed phase MPLC (Interchim C18-HP-Column, H_2O/MeCN, gradient: 20 - 100% MeCN) and freeze-dried to obtain the title compound **27** (6.30 mg, 5.94 µmol, 32%) as a colorless solid.

^{1}H-NMR (400 MHz, MeOH-d_4): δ = 7.50 – 7.44 (m, 2H), 7.41 – 7.31 (m, 10H), 7.26 – 7.18 (m, 8H), 6.92 – 6.85 (m, 2H), 6.74 (dd, *J* = 8.5, 1.8 Hz, 2H), 6.65 (d, *J* = 8.2 Hz, 2H), 5.17 – 5.02 (m, 2H), 4.97 – 4.89 (m, 6H), 4.82 – 4.77 (m, 1H), 4.67 (d, *J* = 10.2 Hz, 2H), 4.58 (d, *J* = 13.3 Hz, 2H), 4.28 (t, *J* = 2.5 Hz, 1H), 4.04 – 3.92 (m, 4H), 3.79 (s, 3H), 3.78 – 3.77 (m, 1H), 3.76 – 3.73 (m, 1H), 3.67 (s, 3H), 3.65 – 3.62 (m, 1H), 3.58 (s, 3H) ppm.

^{31}P{^{1}H}-NMR (162 MHz, MeOH-d_4): δ = - 1.19 (1P), - 11.29 (d, *J* = 13.1 Hz, 1P), - 12.49 (d, *J* = 13.2 Hz, 1P) ppm.

HRMS (ESI) $[M+Na]^+$ calc. for $C_{52}H_{56}O_{18}NaP_3$: 1084.2583, found 1084.2579.

4.5 Synthesis of 5-PP-1/3-InsP$_1$ (**28**)

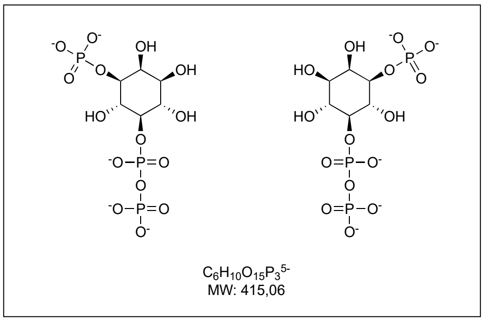

InsP$_3$ intermediate **27** (6.30 mg, 5.94 µmol, 1.0 eq.) was dissolved in MeOH/H_2O (3 mL, 1:1). $NaHCO_3$ (2.49 mg, 29.6 µmol, 5.0 eq.) and Pd/C (10% on activated charcoal, 80.0 mg) were added and the reaction was hydrogenated 4 d at 35 bar (autoclave). The catalyst was removed by filtration and the aqueous phase was extracted with CH_2Cl_2 (2 × 25 mL) and freeze-dried. The crude product was dissolved in H_2O (500 µL) and precipitated by addition of cold acetone (45 mL). Purification was achieved by SAX-column chromatography (Q-Sepharose, HiTrap CaptoQ ImpRes, eluted by NH_4HCO_3-buffer Gradient, 1M). Lyophilization yielded the title compound **28** (eluting at 0.25 to 0.35M buffer concentration, 1.60 mg, 3.85 µmol, 65%) as a colorless solid.

^{1}H-NMR (400 MHz, D_2O): δ = 4.21 (dd, *J* = 3.5, 2.9 Hz, 1H), 4.02 (ddd, *J* = 9.8, 9.8, 9.2 Hz, 1H), 3.92 (ddd, *J* = 9.8, 9.8, 2.9 Hz, 1H), 3.82 (dd, *J* = 9.8, 9.2 Hz, 1H) 3.74 (dd, *J* = 9.8, 9.2 Hz, 1H), 3.56 (dd, *J* = 9.8, 2.9 Hz, 1H) ppm.

$^{31}P\{^1H\}$-NMR (162 MHz, D_2O): δ = 3.22 (1P), - 5.69 (d, *J* = 17.0 Hz, 1P), - 9.92 (d, *J* = 17.2 Hz, 1P) ppm.

^{13}C-NMR (101 MHz, D2O): δ = 80.25 (d, *J* = 7.0 Hz), 73.62, 71.41, 71.21 (2 ×C), 70.32 ppm.

HRMS (ESI) $[M-H]^-$ calc. for $C_6H_{14}O_{15}P_3$: 418.9551, found 418.9551.

7. Literature

1. Jessen, H.J.; Schulz, T.; Balzarini, J.; Meier, C. Bioreversible Protection of Nucleoside Diphosphates. *Angew. Chem. Int. Ed.* **2008**, *47*, 8719–8722, doi:10.1002/anie.200803100.
2. Hostachy, S.; Utesch, T.; Franke, K.; Dornan, G.L.; Furkert, D.; Türkaydin, B.; Haucke, V.; Sun, H.; Fiedler, D. Dissecting the Activation of Insulin Degrading Enzyme by Inositol Pyrophosphates and Their Bisphosphonate Analogs. *Chem. Sci.* **2021**, *12*, 10696–10702, doi:10.1039/D1SC02975D.
3. Zhang, H.; Thompson, J.; Prestwich, G.D. A Scalable Synthesis of the IP_7 Isomer, 5-PP-Ins(1,2,3,4,6)P_5. *Org. Lett.* **2009**, *11*, 1551–1554, doi:10.1021/ol900149x.
4. Das, S.; Parekh, N.; Mondal, B.; Gupta, S. Sen Controlled Synthesis of End-Functionalized Mannose-6-Phosphate Glycopolypeptides for Lysosome Targeting. *ACS Macro Lett.* **2016**, *5*, 809–813, doi:10.1021/acsmacrolett.6b00297.
5. Capolicchio, S.; Thakor, D.T.; Linden, A.; Jessen, H.J. Synthesis of Unsymmetric Diphospho-Inositol Polyphosphates. *Angew. Chem. Int. Ed.* **2013**, *52*, 6912–6916, doi:10.1002/anie.201301092.
6. Haas, T.M.; Mundinger, S.; Qiu, D.; Jork, N.; Ritter, K.; Dürr-Mayer, T.; Ripp, A.; Saiardi, A.; Schaaf, G.; Jessen, H.J. Stable Isotope Phosphate Labelling of Diverse Metabolites Is Enabled by a Family of ^{18}O-Phosphoramidites. *Angew. Chem. Int. Ed.* **2022**, *61*, e202112457, doi:10.1002/anie.202112457.
7. Capolicchio, S.; Wang, H.; Thakor, D.T.; Shears, S.B.; Jessen, H.J. Synthesis of Densely Phosphorylated Bis-1,5-Diphospho-Myo-Inositol Tetrakisphosphate and Its Enantiomer by Bidirectional P-Anhydride Formation. *Angew. Chem. Int. Ed.* **2014**, *53*, 9508–9511, doi:10.1002/anie.201404398.
8. Kiely, D.E.; Abruscato, G.J.; Baburao, V. A Synthesis of (±)Myo-Inositol 1-Phosphate. *Carbohydr. Res.* **1974**, *34*, 307–313, doi:10.1016/S0008-6215(00)82905-5.
9. Subramanian, D.; Laketa, V.; Müller, R.; Tischer, C.; Zarbakhsh, S.; Pepperkok, R.; Schultz, C. Activation of Membrane-Permeant Caged PtdIns(3)P Induces Endosomal Fusion in Cells. *Nat. Chem. Biol.* **2010**, *6*, 324–326, doi:10.1038/nchembio.348.
10. Rukavishnikov, A. V; Ryan, M.; Griffith, O.H.; Keana, J.F.W. A Chromogenic Substrate for the Continuous Assay of Mammalian Phosphoinositide-Specific Phospholipase C. *Bioorg. Med. Chem. Lett.* **1997**, *7*, 1239–1242, doi:10.1016/S0960-894X(97)00198-4.

Protected Inositol Derivative **11**, ^{1}H - NMR ($CDCl_3$, 400 MHz)

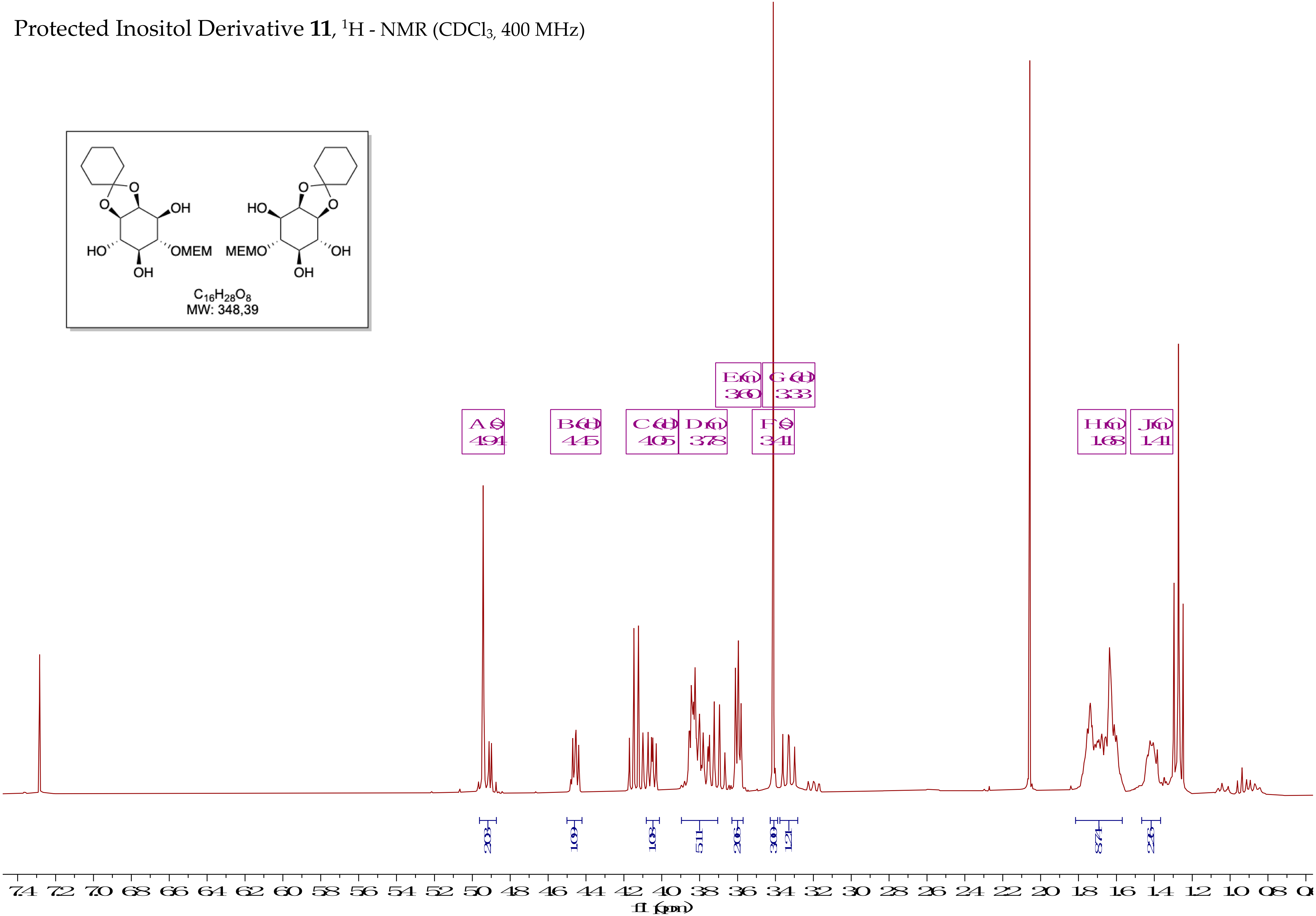

Protected Inositol Derivative **11**, ^{13}C - NMR ($CDCl_3$, 101 MHz)

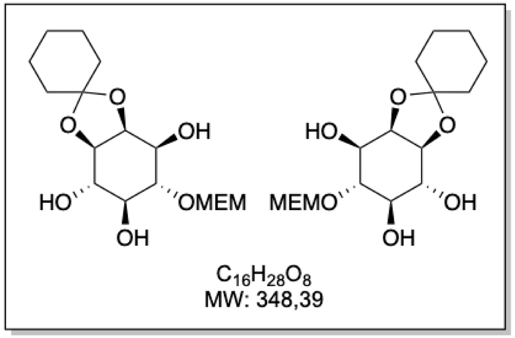

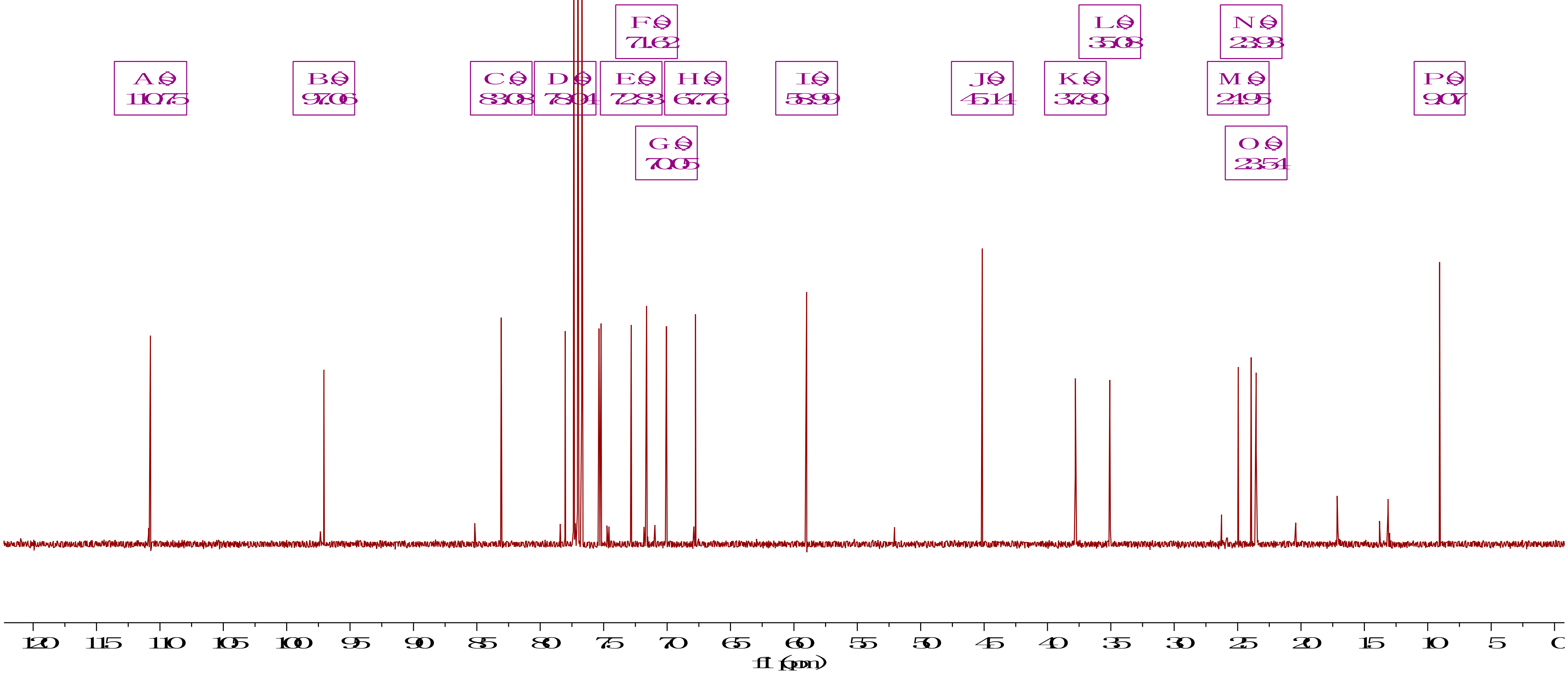

Protected InsP$_3$ Derivative **12**, ^{1}H - NMR ($CDCl_3$, 400 MHz)

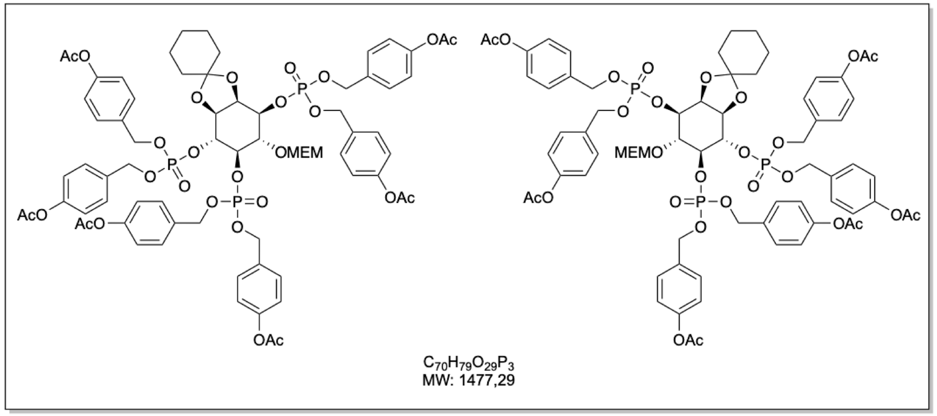

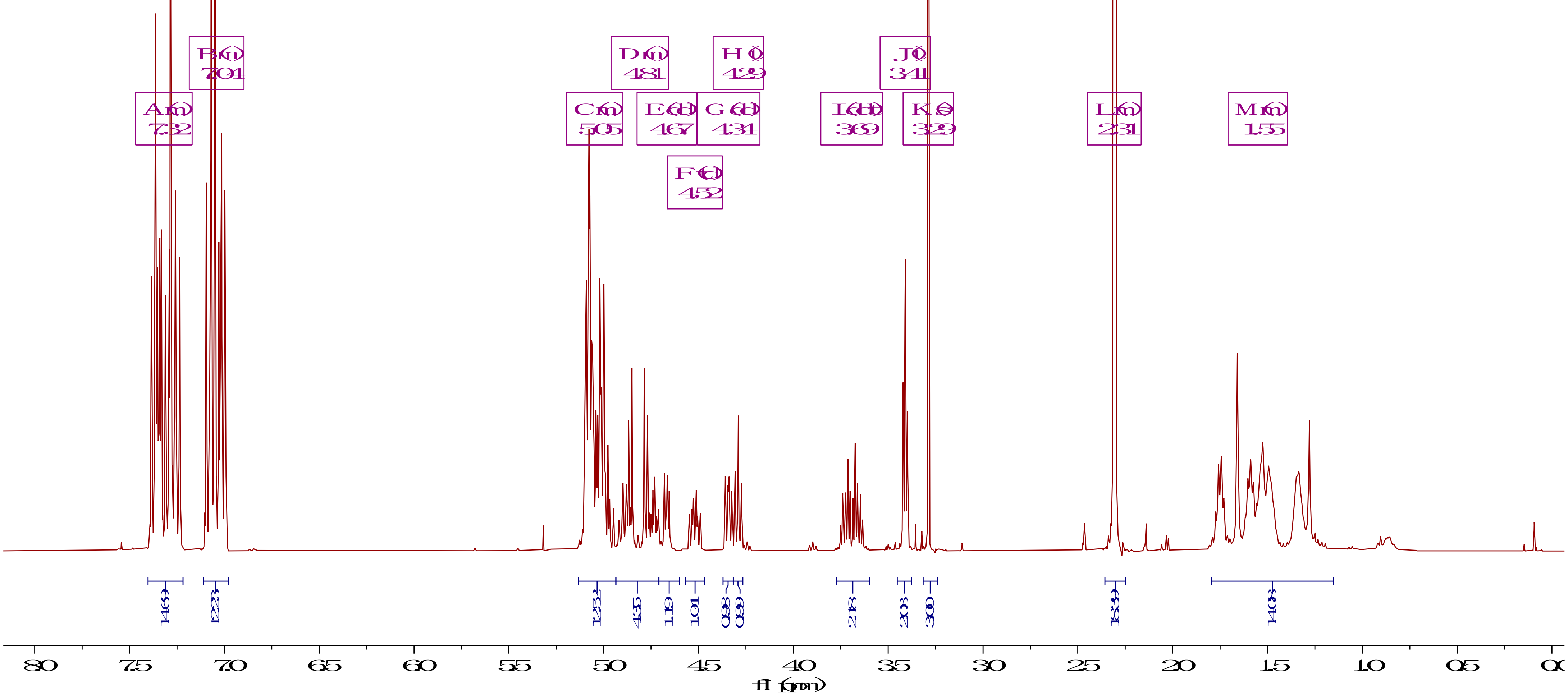

Protected $InsP_3$ Derivative **12**, $^{31}P\{^{1}H\}$ - NMR ($CDCl_3$, 162 MHz)

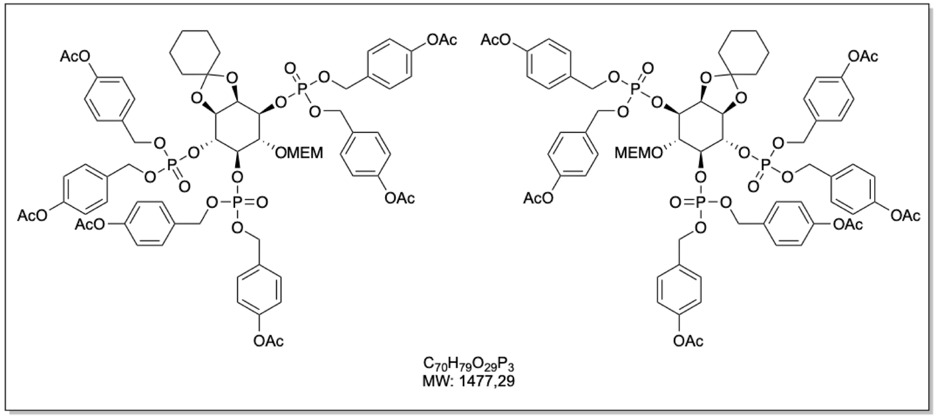

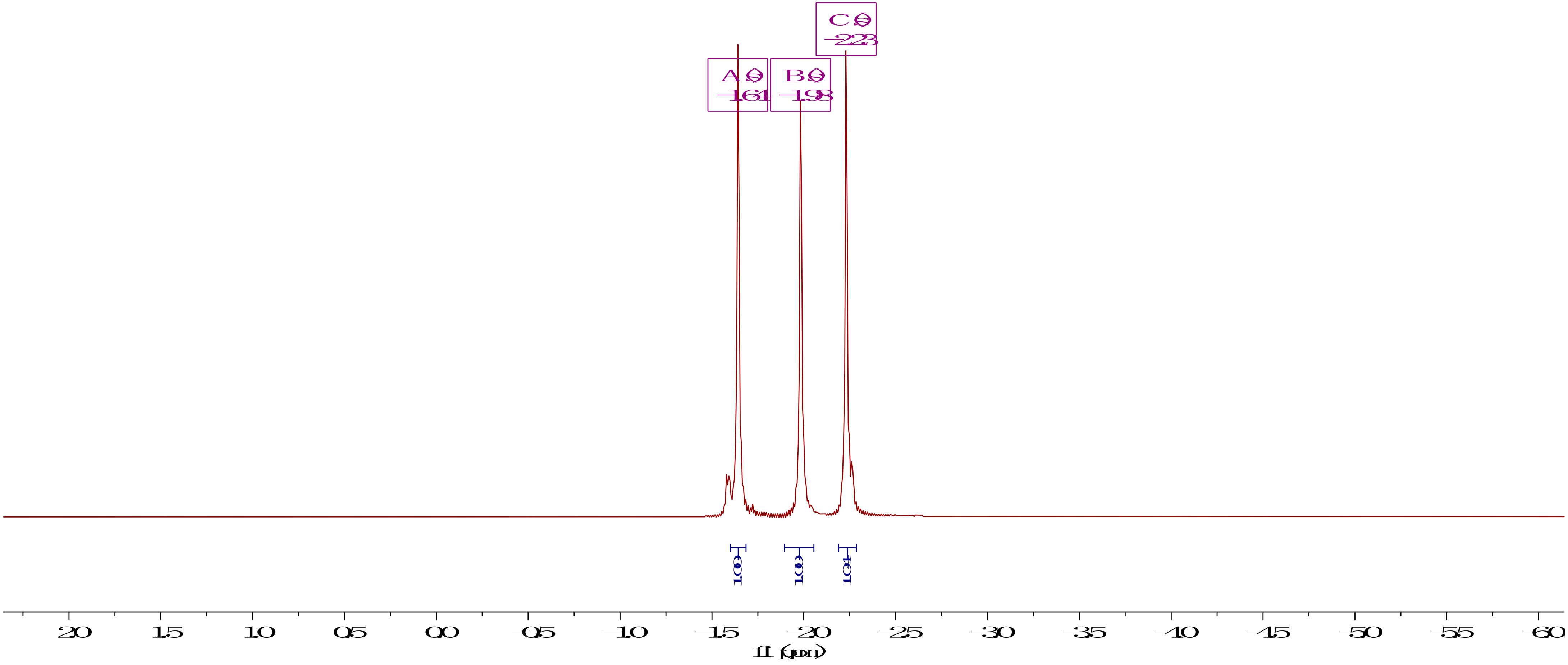

Protected $InsP_3$ Derivative **12**, ^{13}C - NMR ($CDCl_3$, 101 MHz)

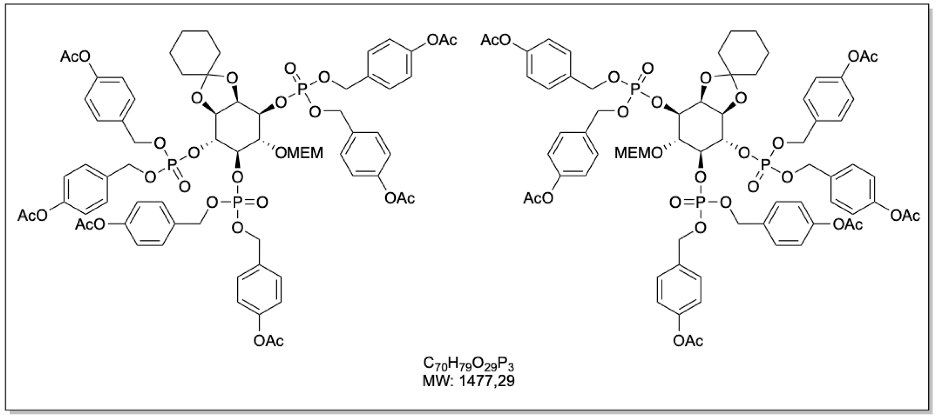

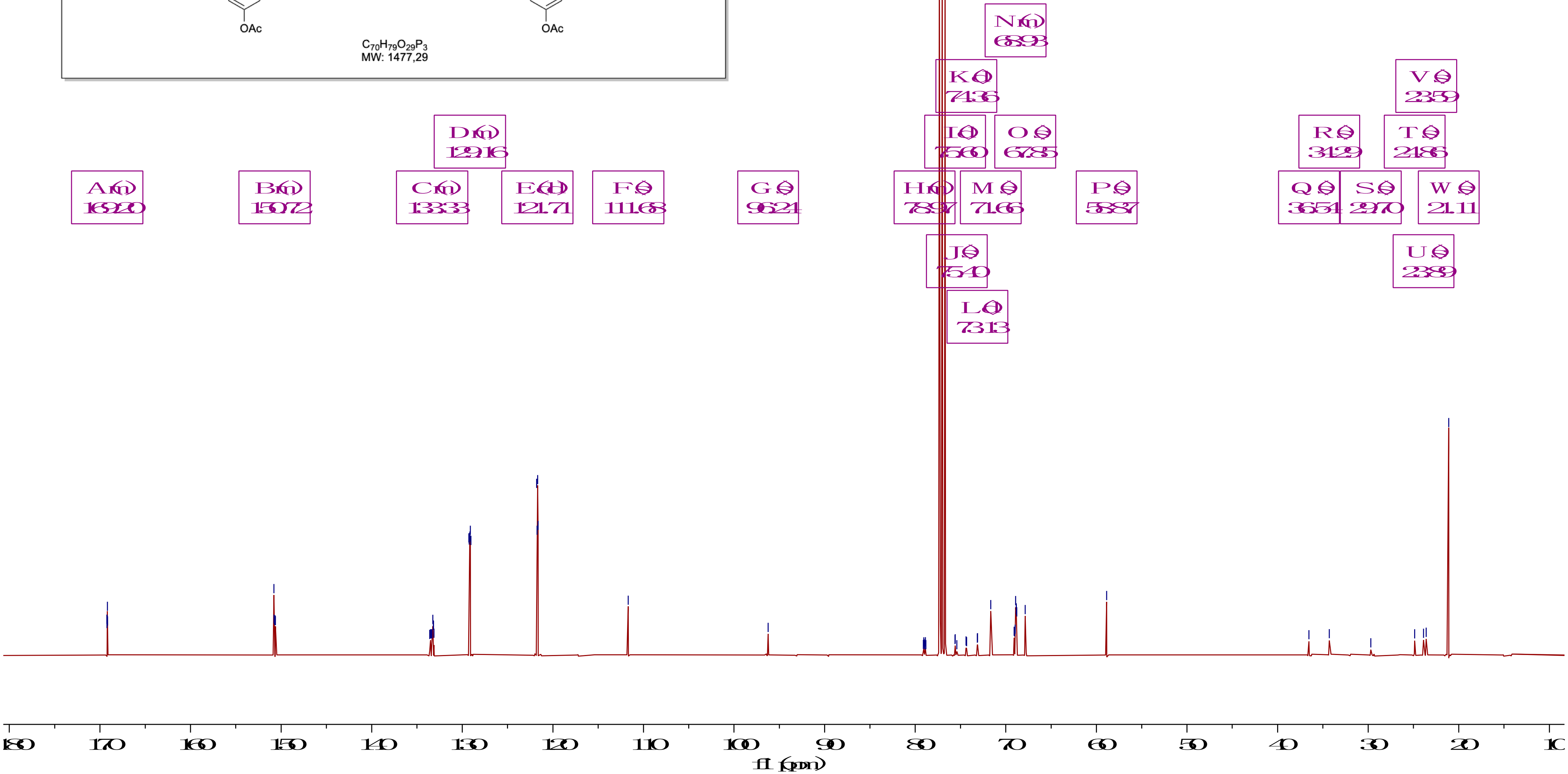

rac-1,4,5-InsP$_3$ (**14**), ^{1}H - NMR (D$_2$O, 400 MHz)

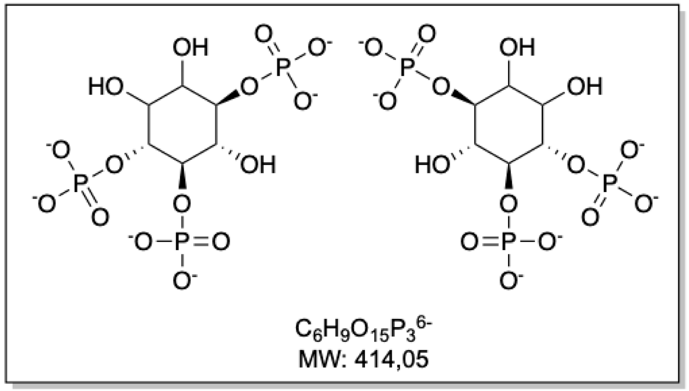

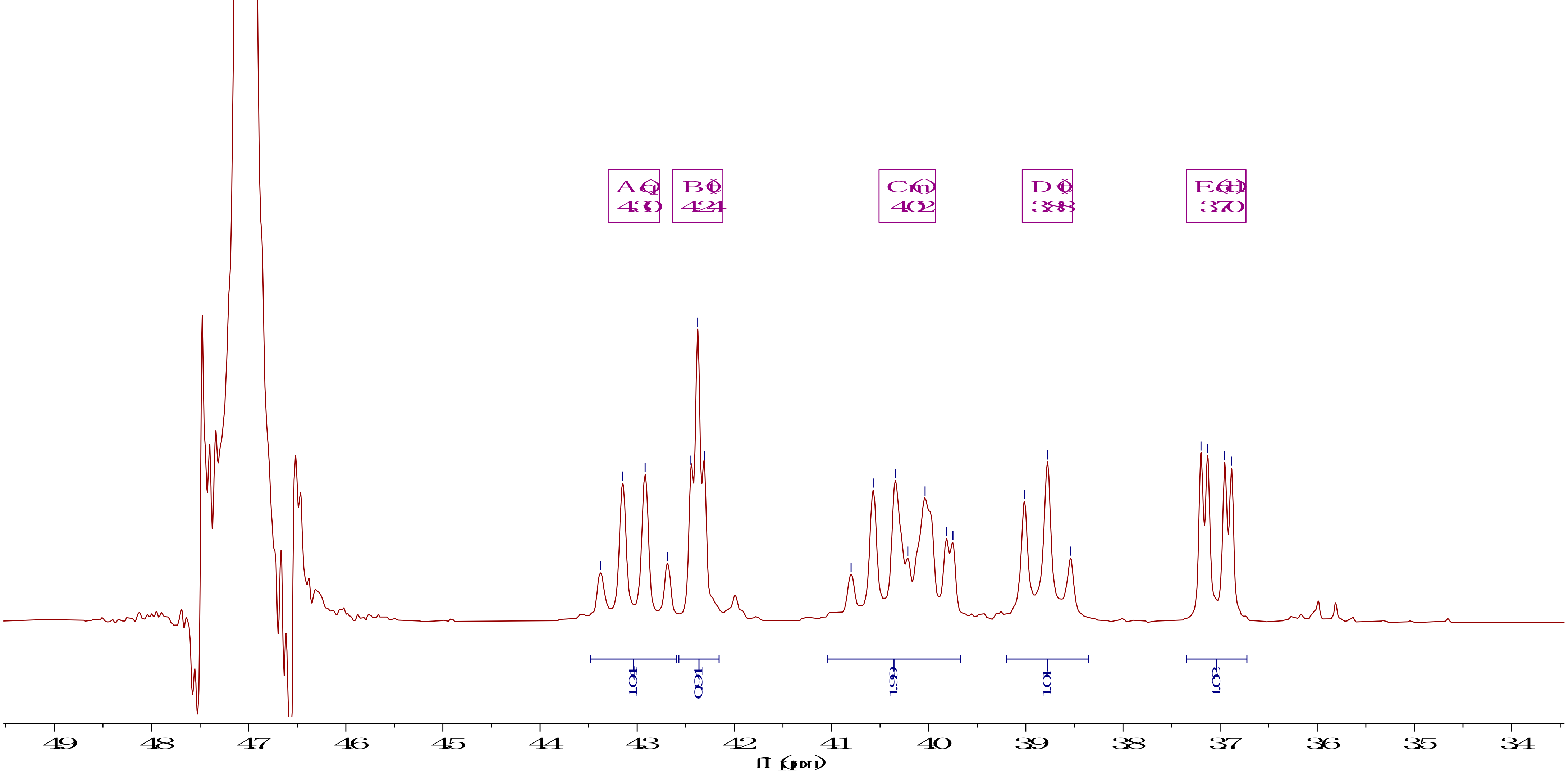

rac-1,4,5-InsP$_3$ (**14**), $^{31}P\{^1H\}$ - NMR (D_2O, 162 MHz)

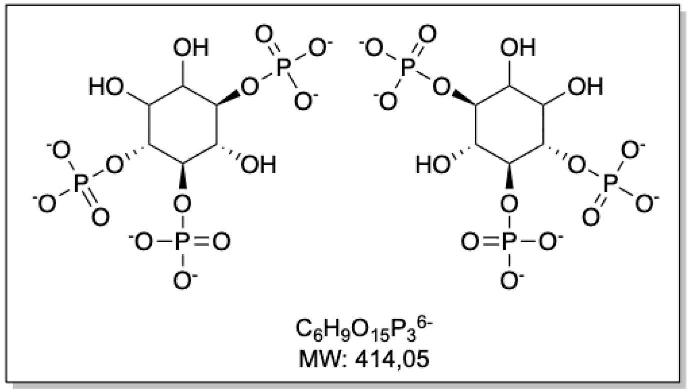

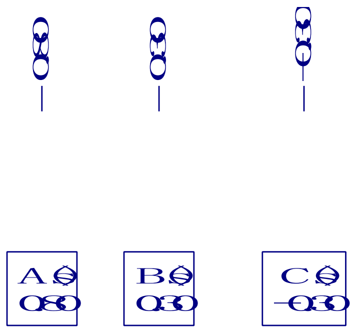

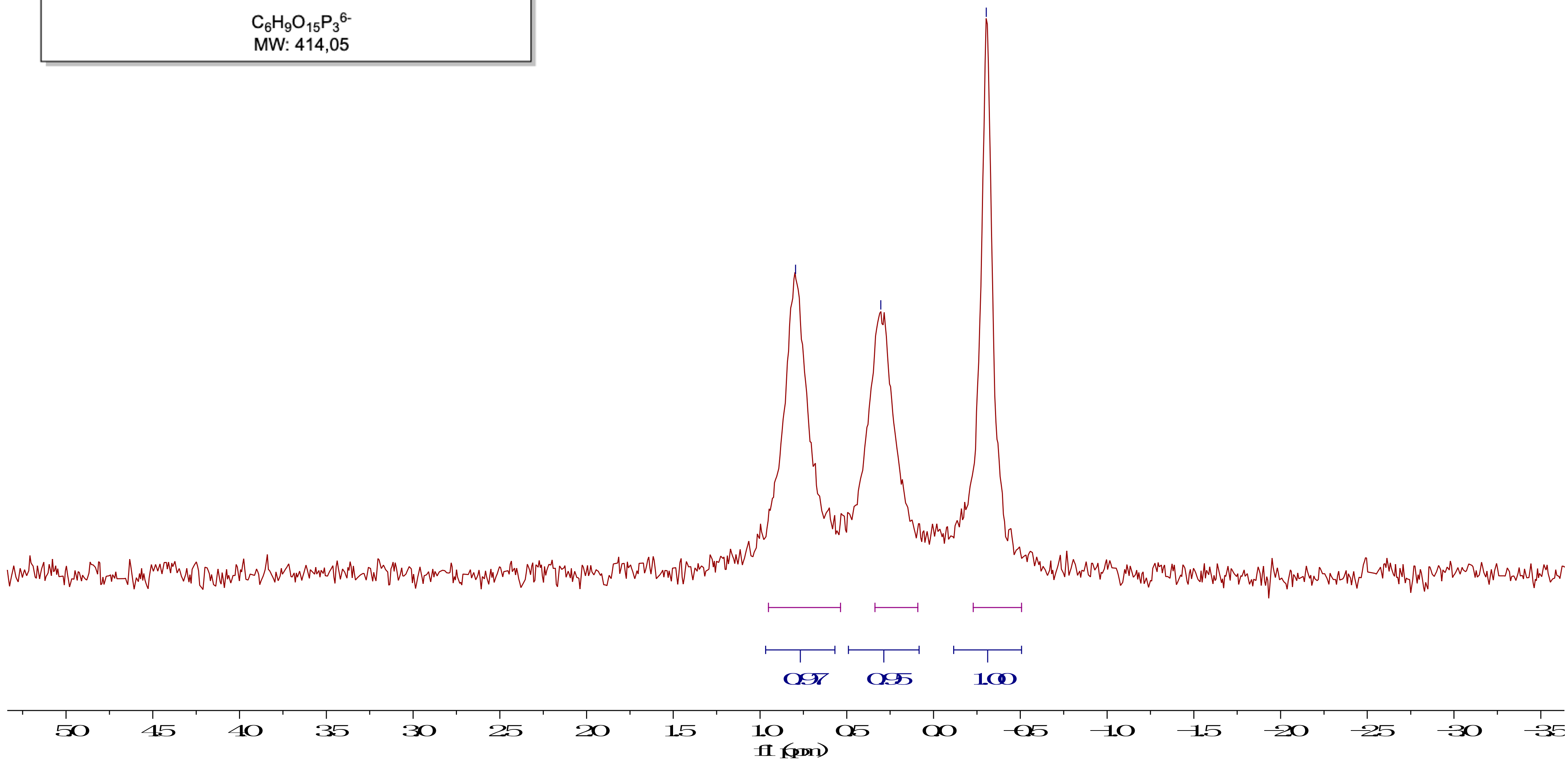

rac-1,4,5-$InsP_3$ (**14**), ^{13}C - NMR (D_2O, 101 MHz)

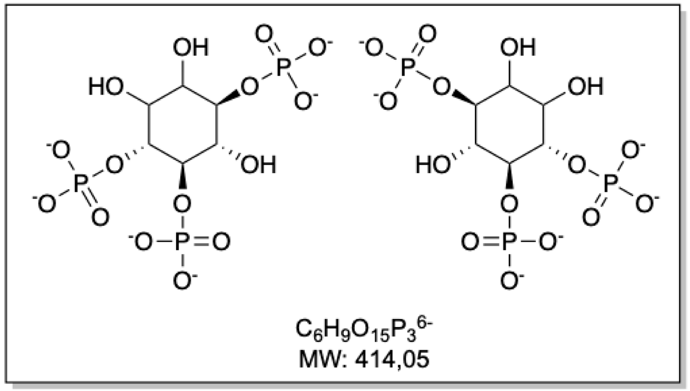

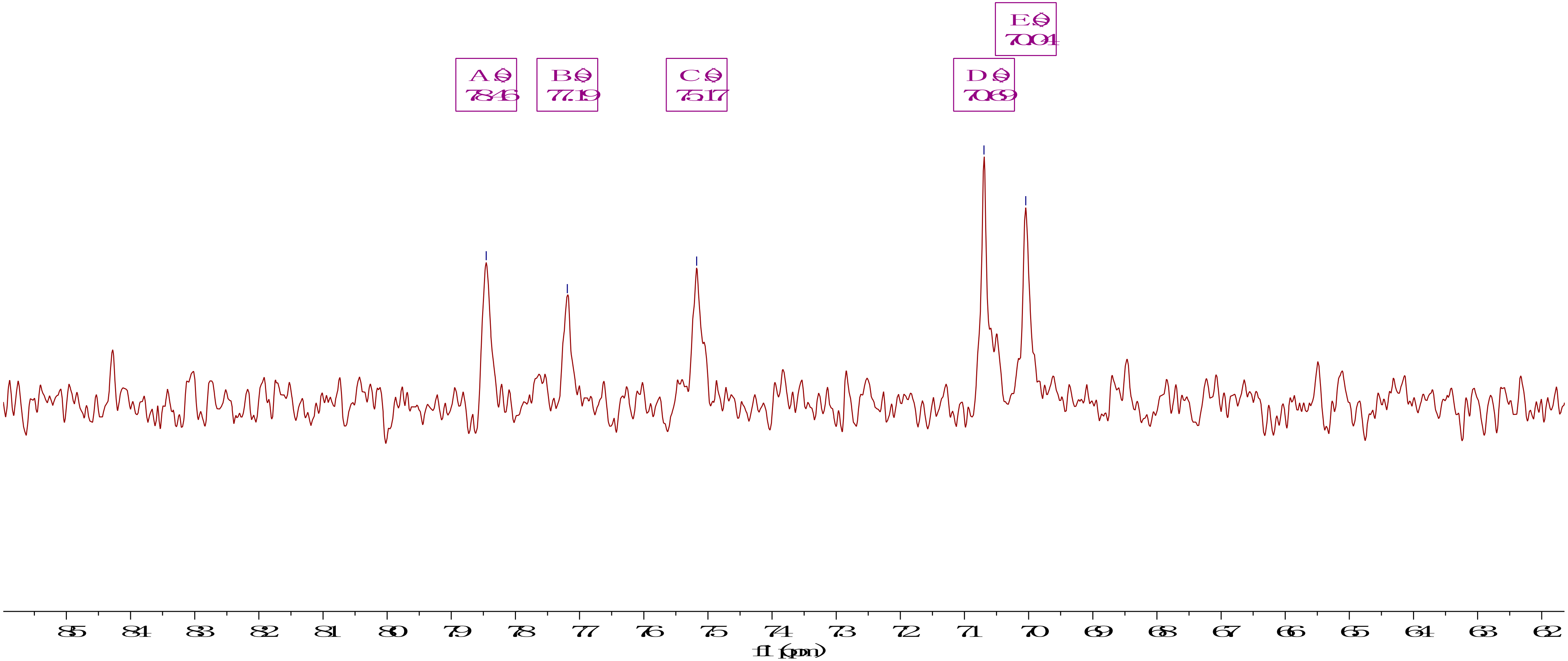

InsP$_4$ derivative **16**, ^{1}H - NMR ($CDCl_3$, 400 MHz)

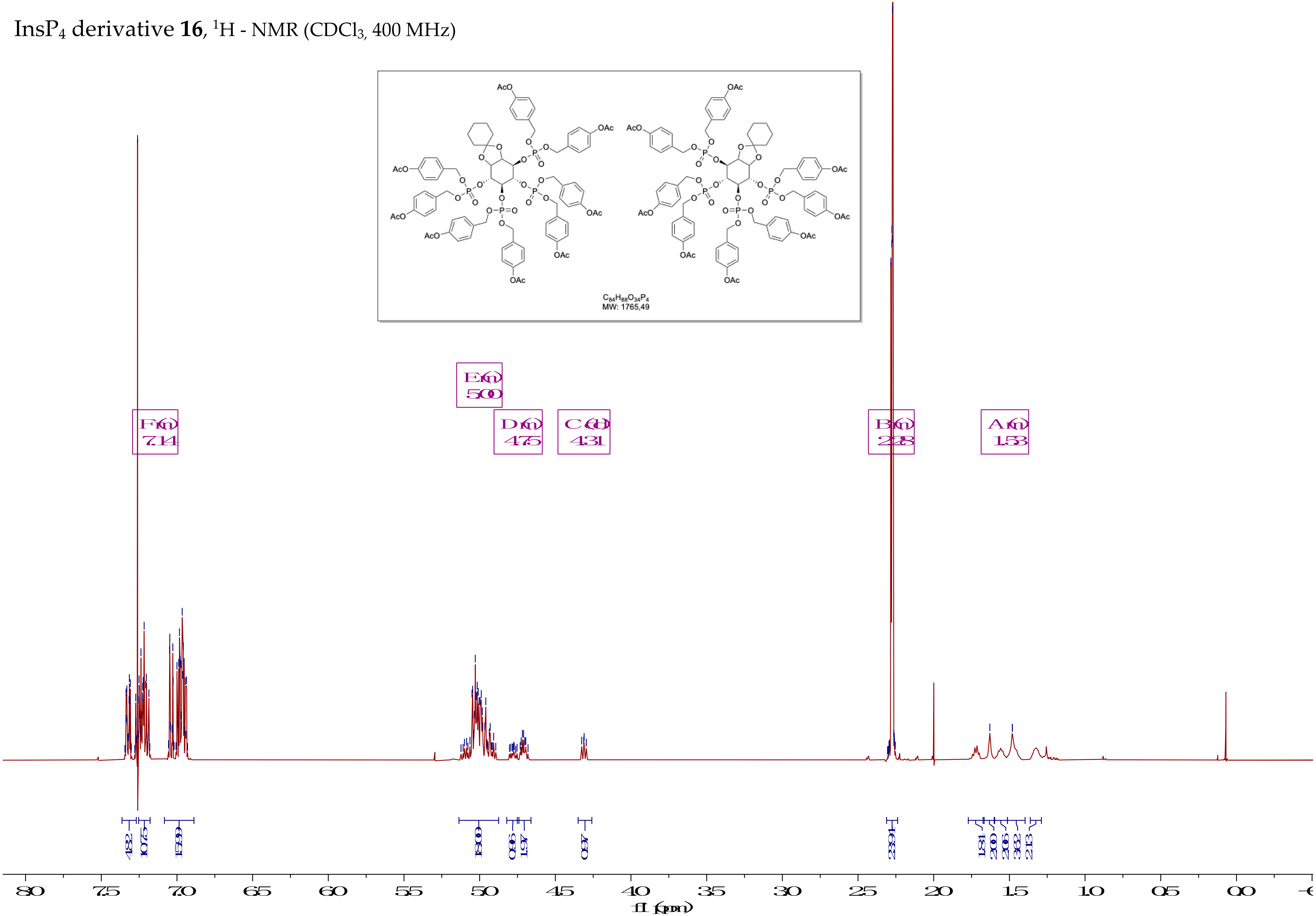

InsP$_4$ derivative **16**, ^{31}P{^{1}H} - NMR ($CDCl_3$, 162 MHz)

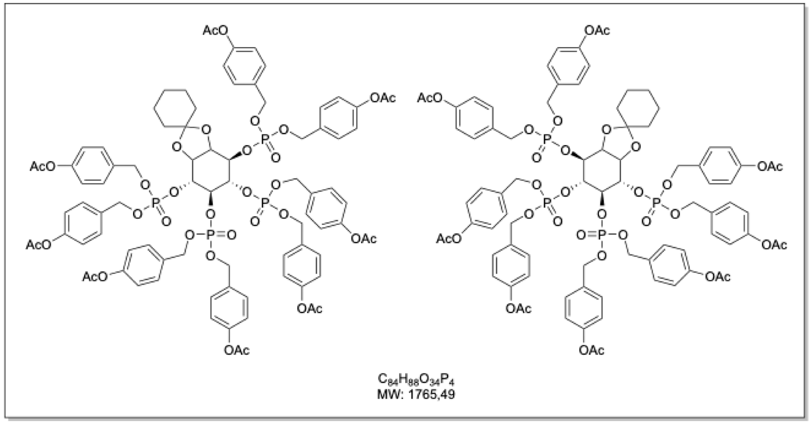

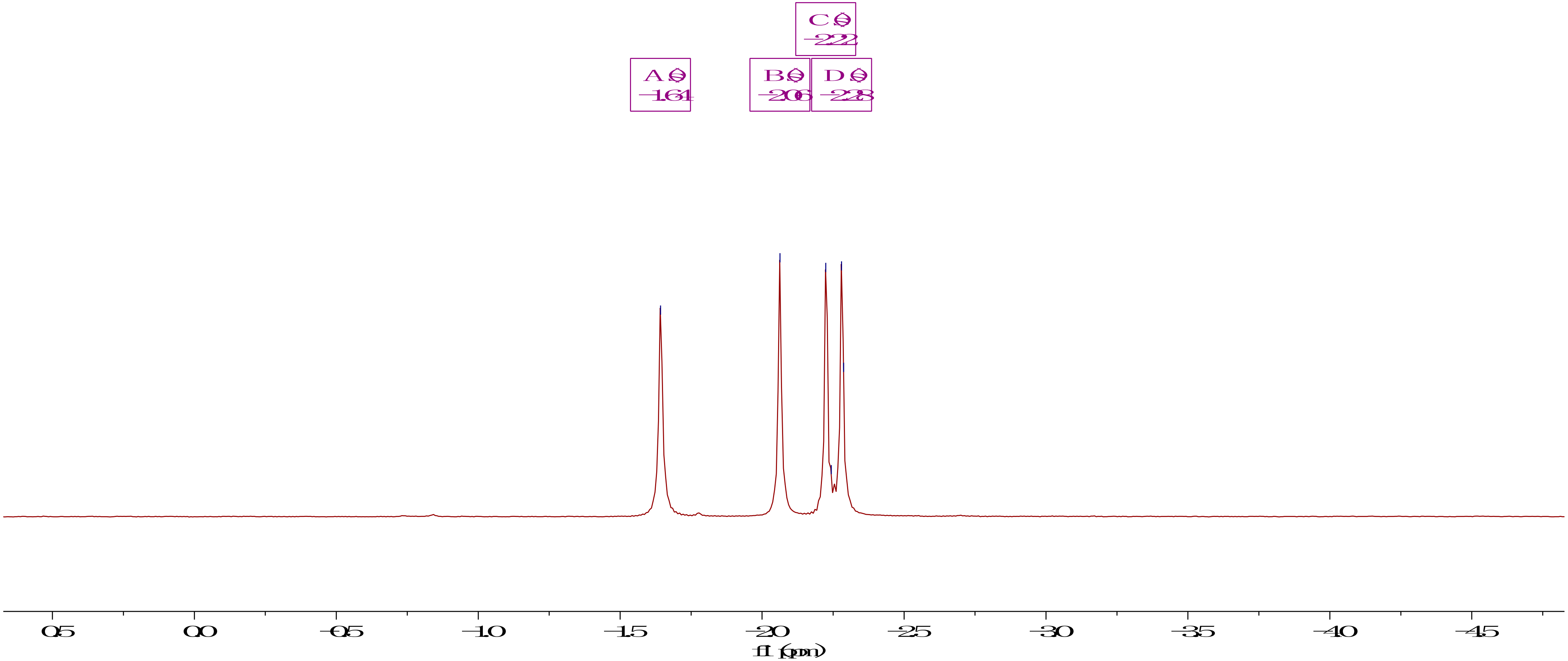

InsP$_4$ derivative **16**, ^{13}C - NMR (CDCl$_3$, 101 MHz)

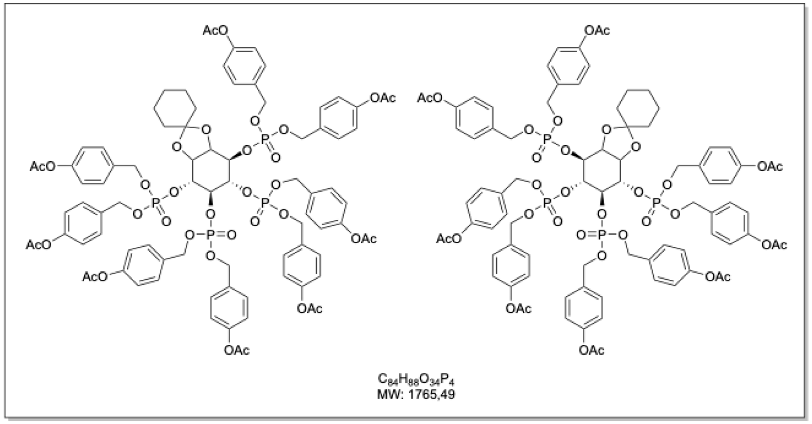

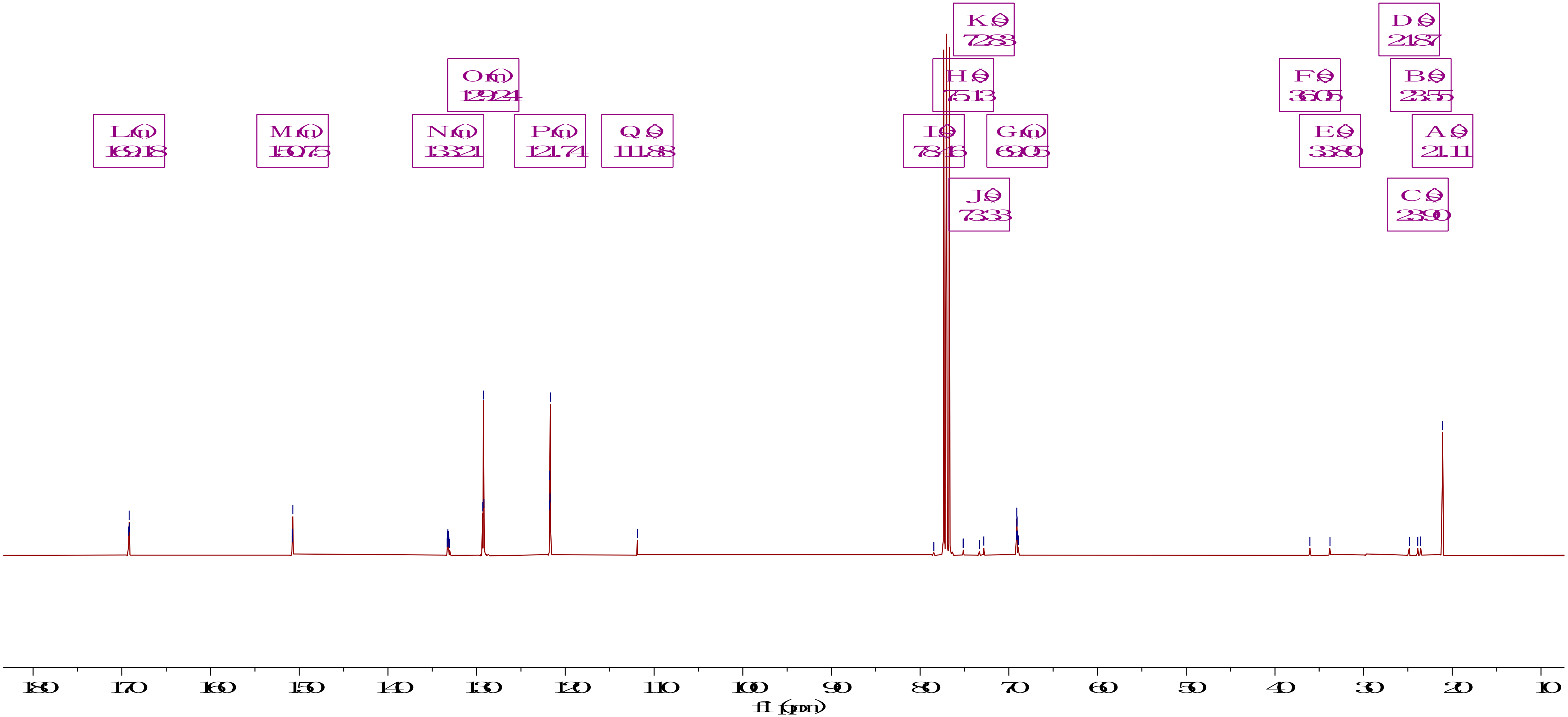

InsP$_4$ derivative **17**, ^{1}H - NMR (D_2O, 400 MHz)

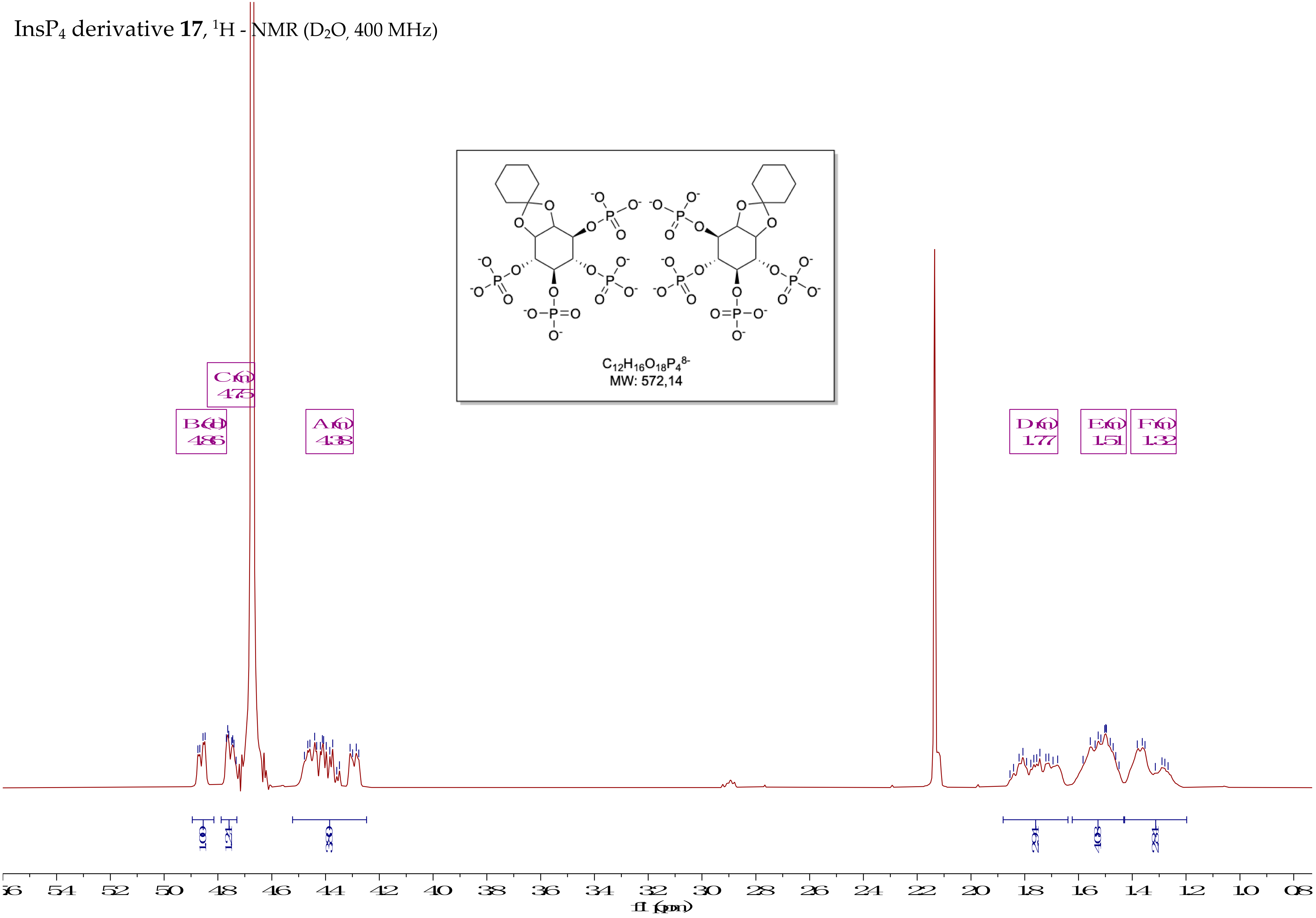

InsP$_4$ derivative **17**, ^{31}P{^{1}H} - NMR (D$_2$O, 162 MHz)

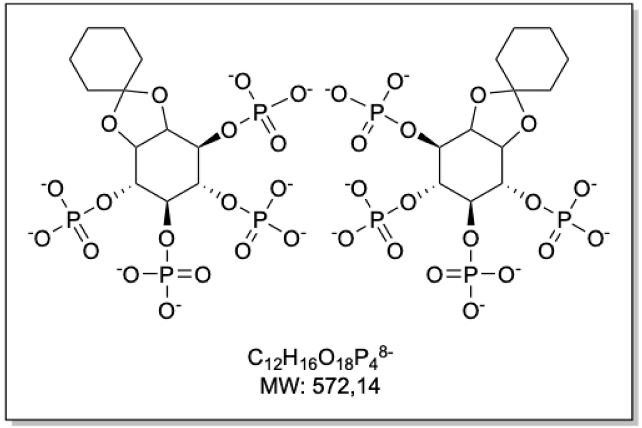

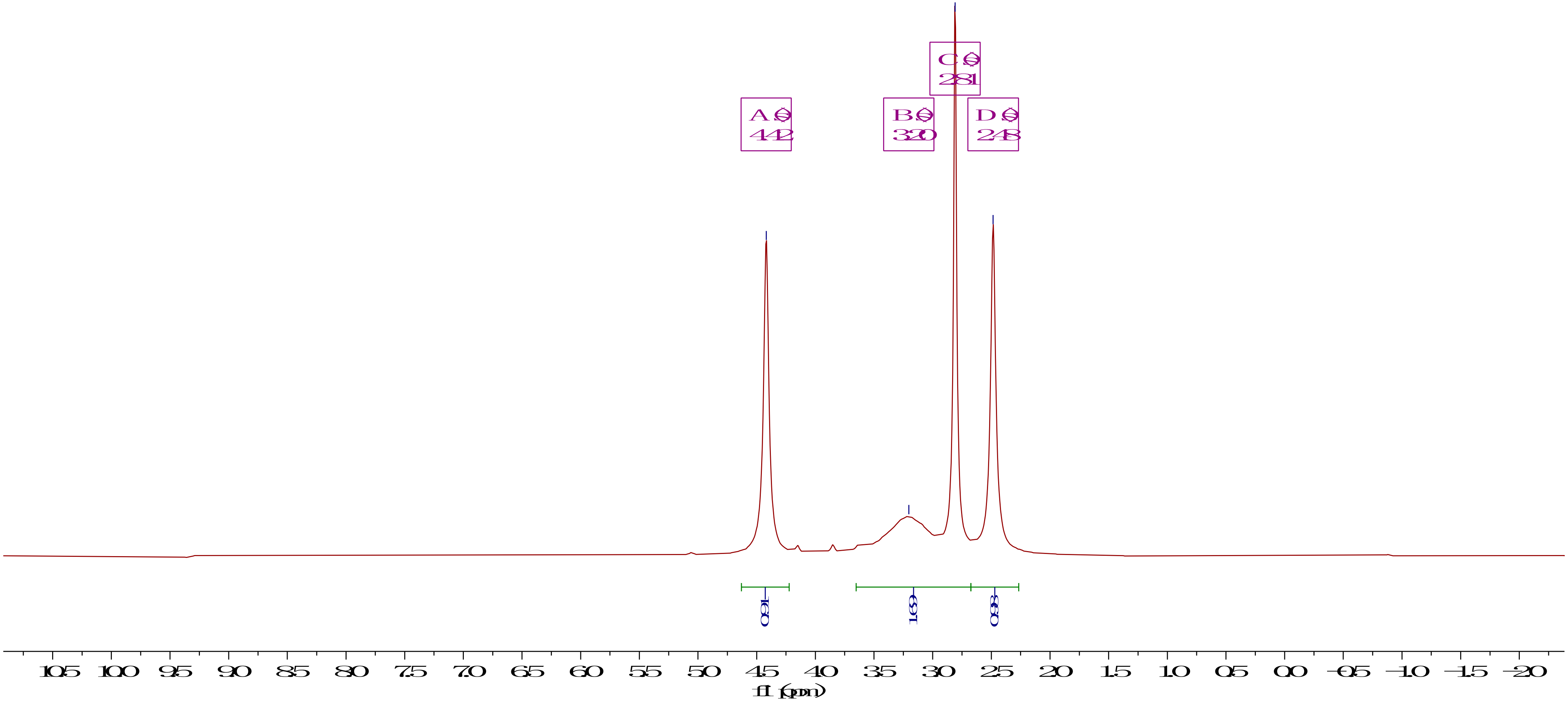

InsP$_4$ derivative **17**, ^{13}C - NMR (D$_2$O, 101 MHz)

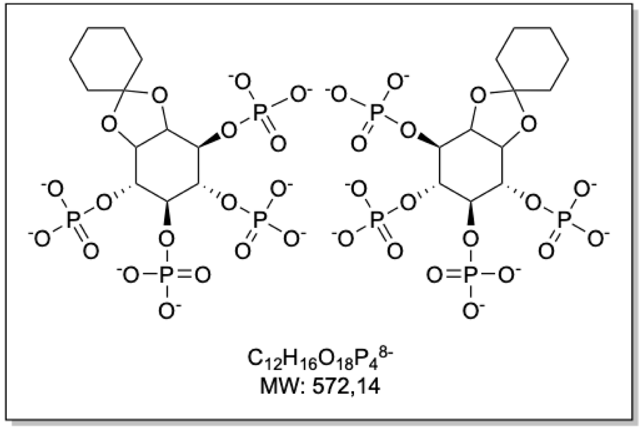

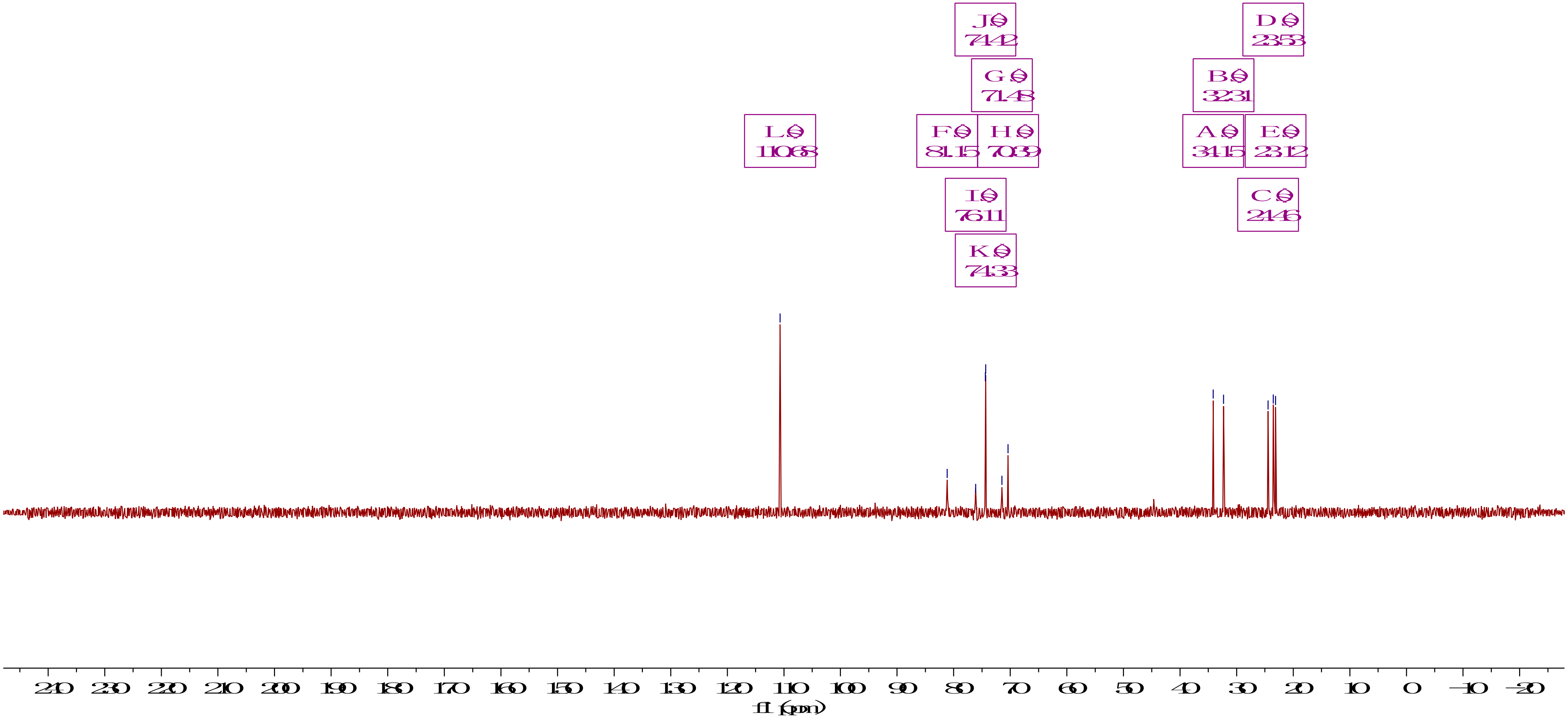

rac-1,4,5,6-InsP$_4$ (**18**), ^{1}H - NMR (D_2O, 400 MHz)

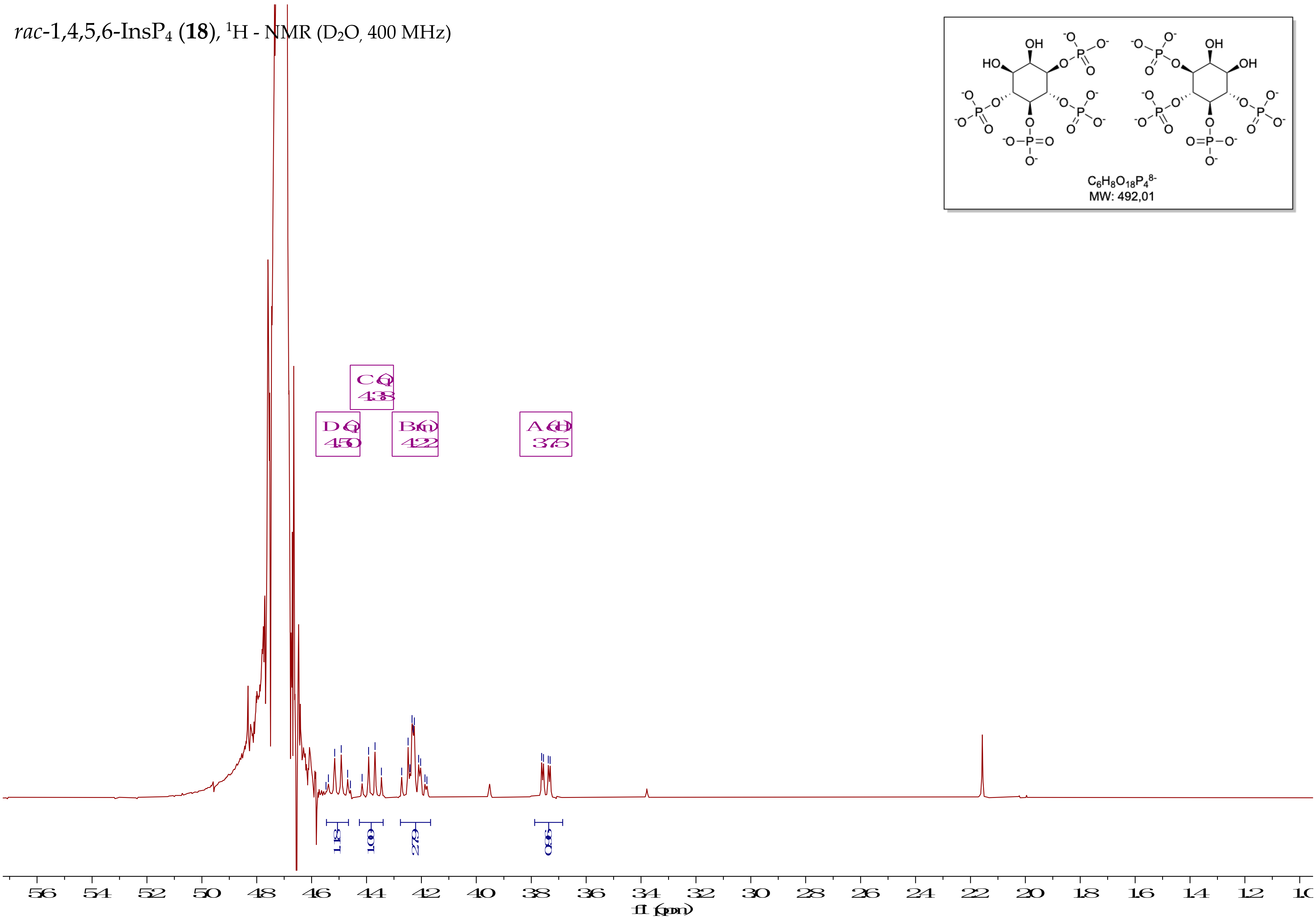

rac-1,4,5,6-$InsP_4$ (**18**), $^{31}P\{^{1}H\}$ - NMR (D_2O, 162 MHz)

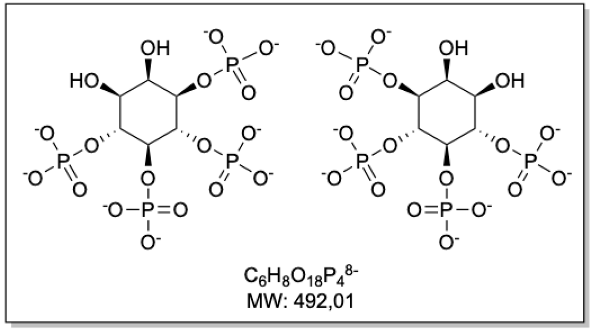

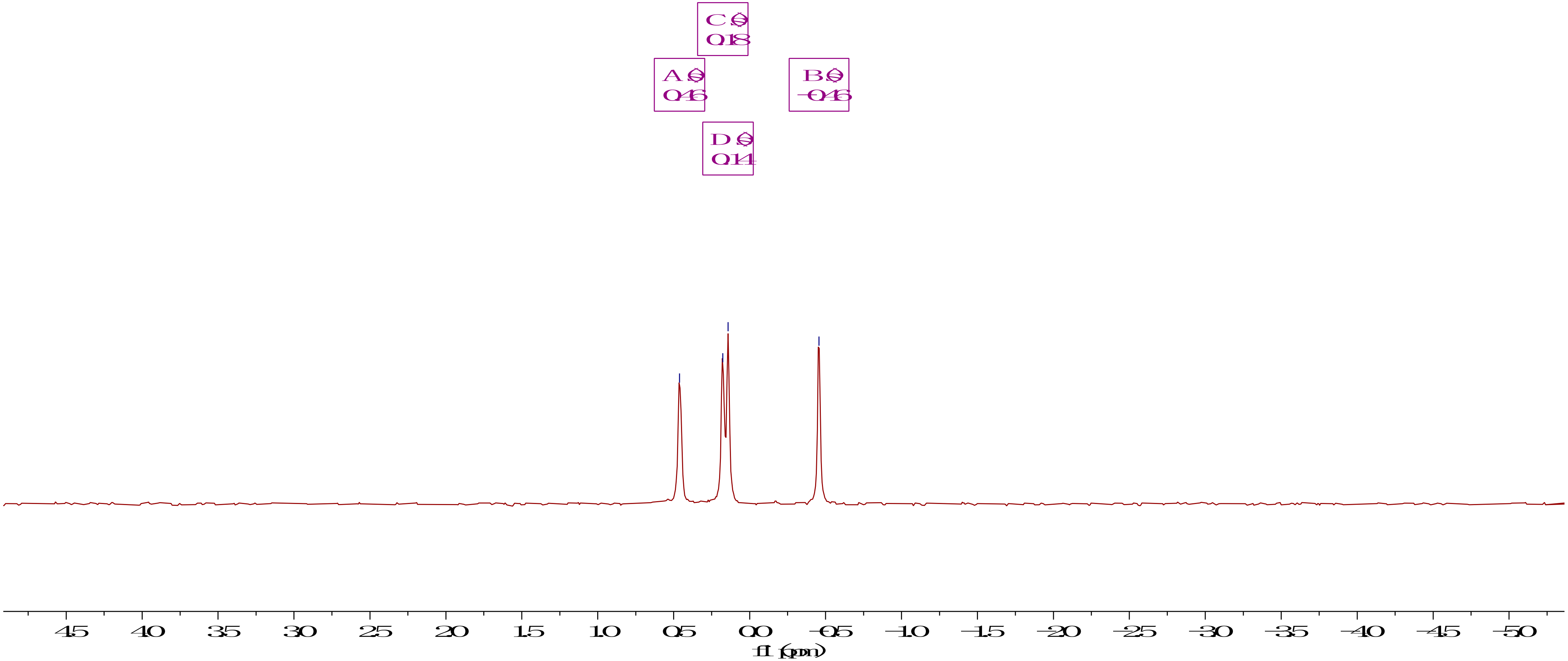

rac-1,4,5,6-InsP$_4$ (**18**), ^{13}C - NMR (D$_2$O, 101 MHz)

(Spectrum contains TFA)

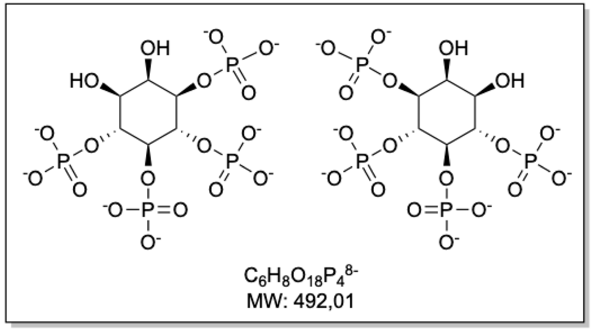

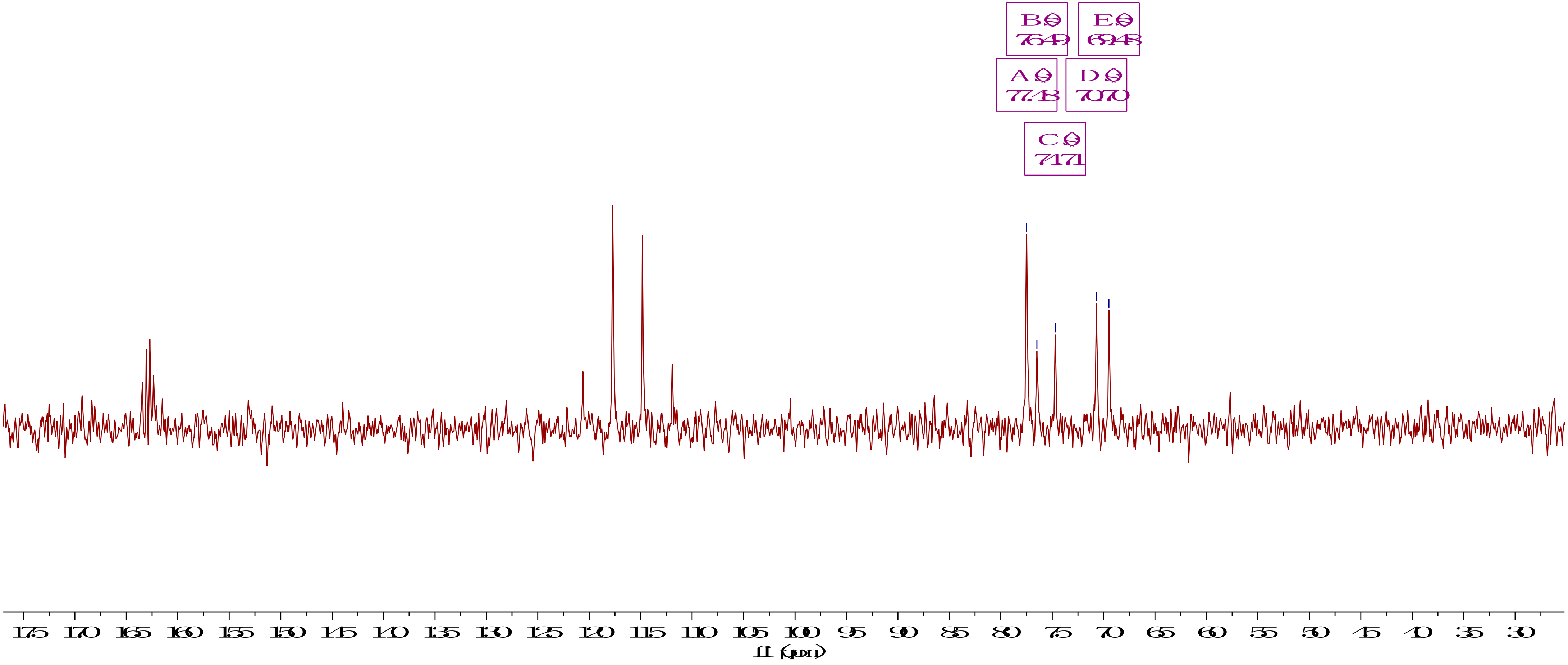

PCP-Derivative **20**, ^{1}H - NMR ($CDCl_3$, 400 MHz)

(contains a mixture of diastereomers)

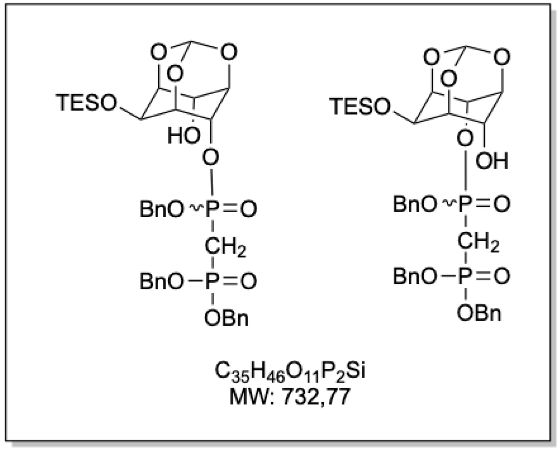

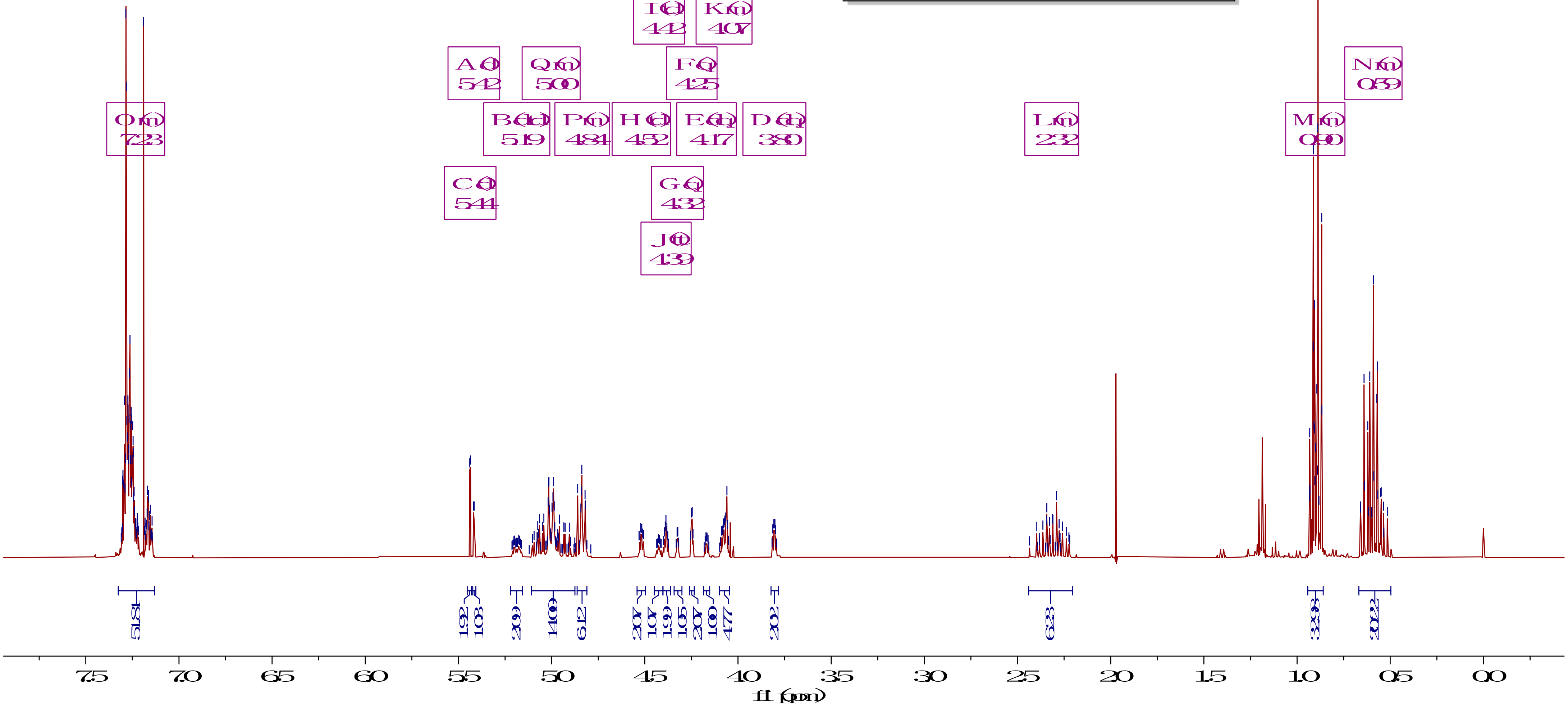

PCP-Derivative **20**, $^{31}P\{^{1}H\}$ - NMR ($CDCl_3$, 162 MHz)

(contains a mixture of diastereomers)

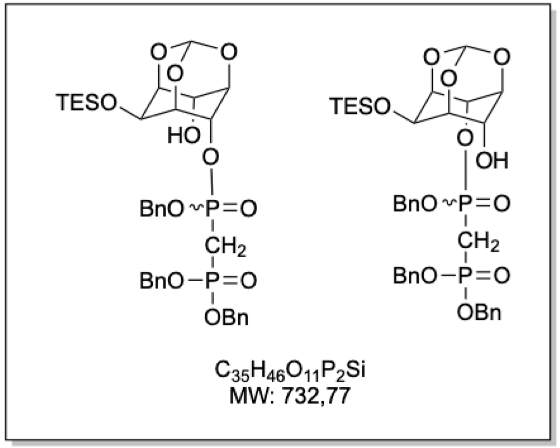

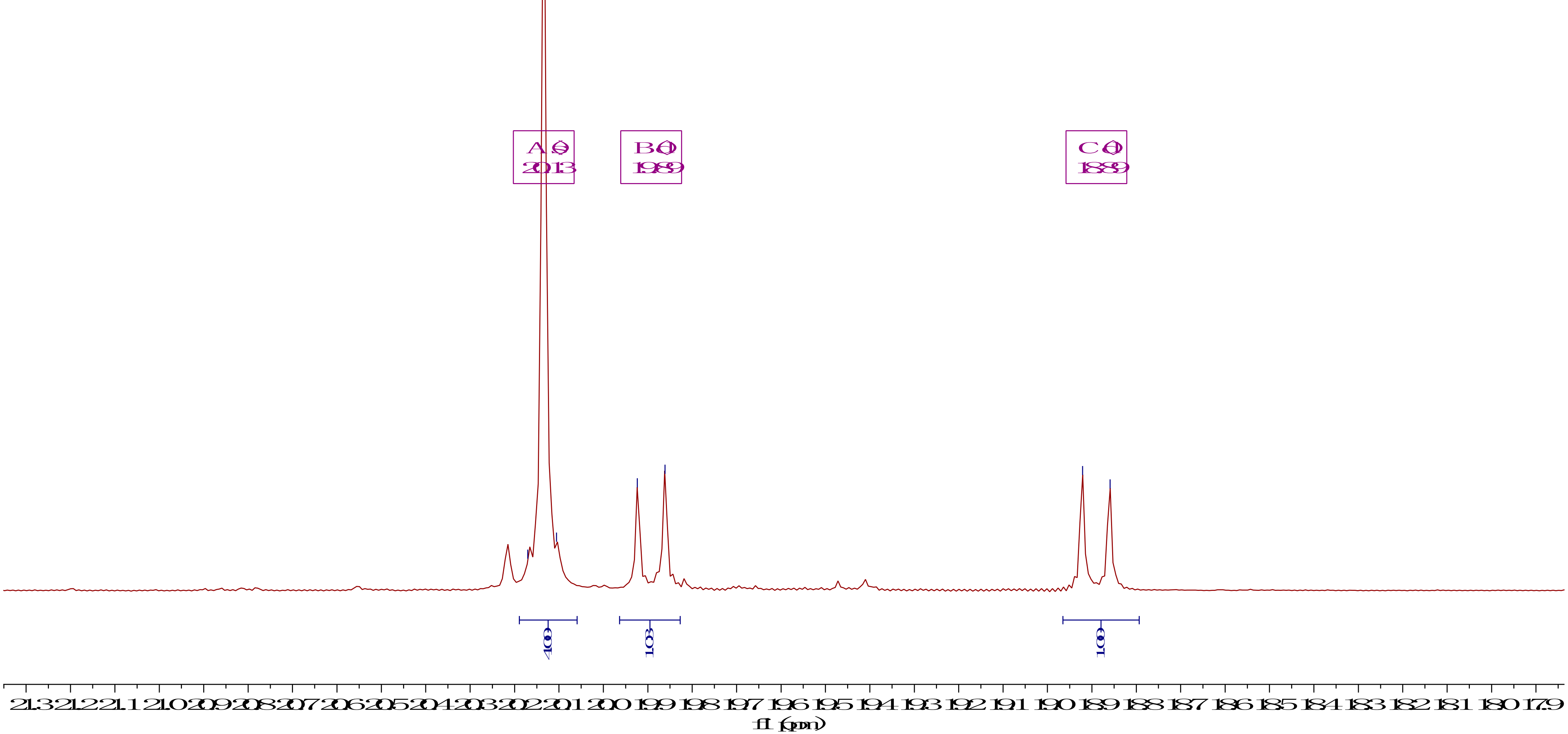

Protected PCP-InsP$_5$ derivative **22**, ^{1}H - NMR ($CDCl_3$, 400 MHz)

(contains a mixture of diastereomers)

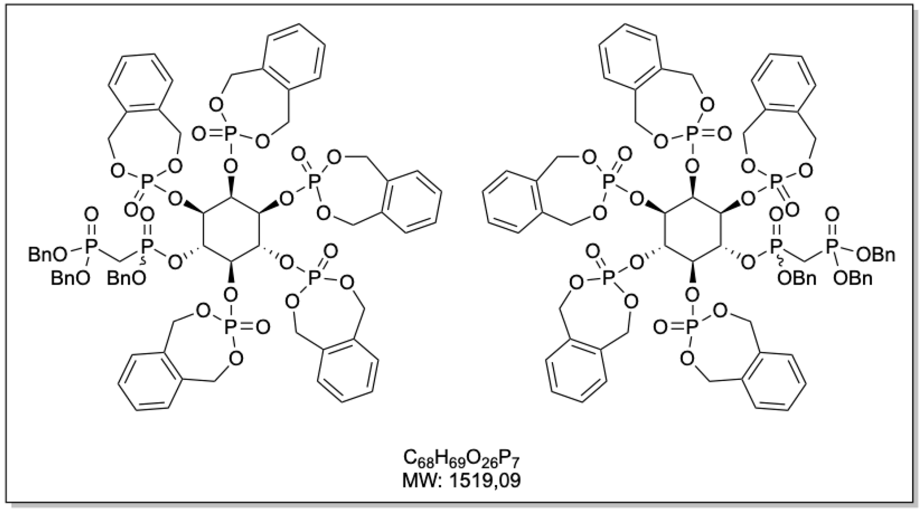

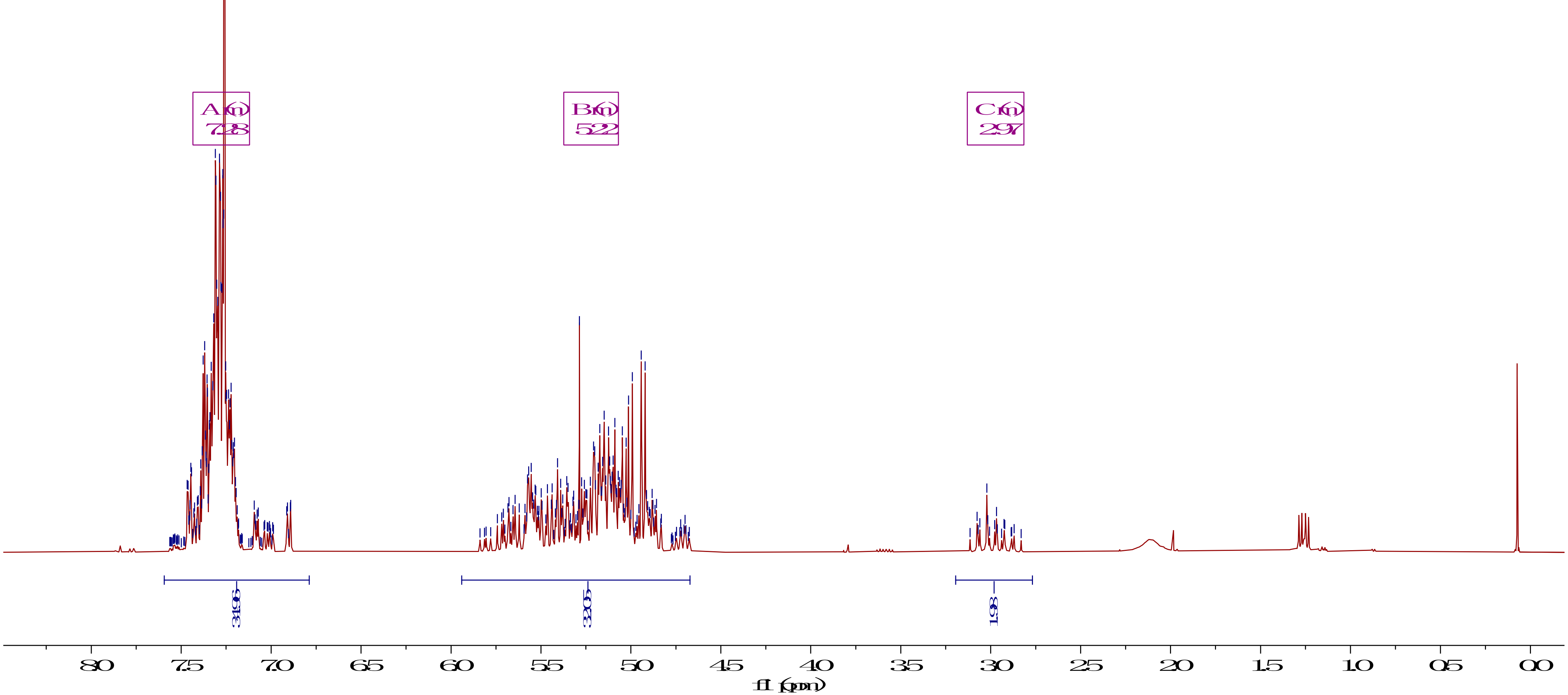

Protected PCP-InsP$_5$ derivative **22**, $^{31}P\{^1H\}$ - NMR ($CDCl_3$, 162 MHz)

(contains a mixture of diastereomers)

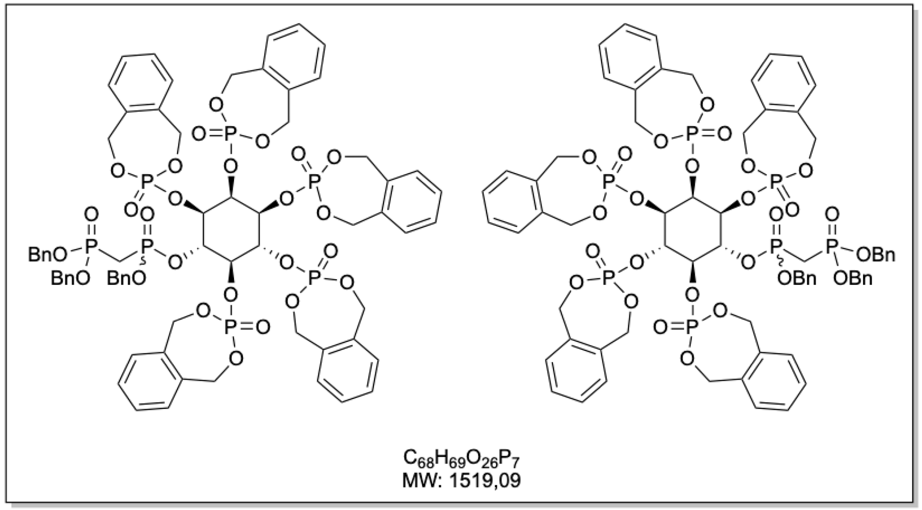

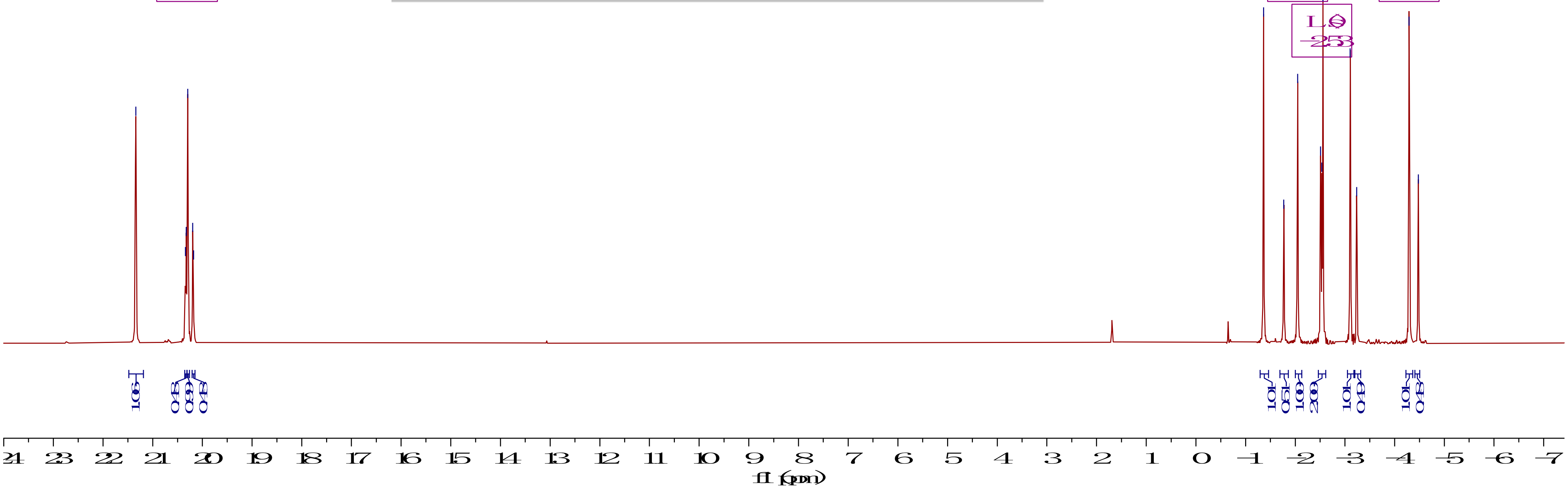

4,6-PCP-InsP$_5$ (**23**), ^{1}H - NMR (D_2O, 400 MHz)

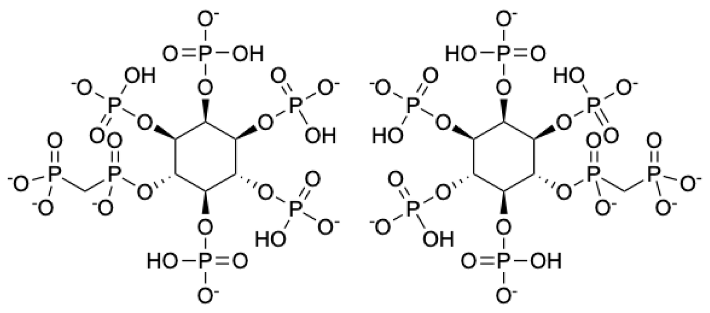

$C_7H_{13}O_{26}P_7^{8-}$
MW: 729,98

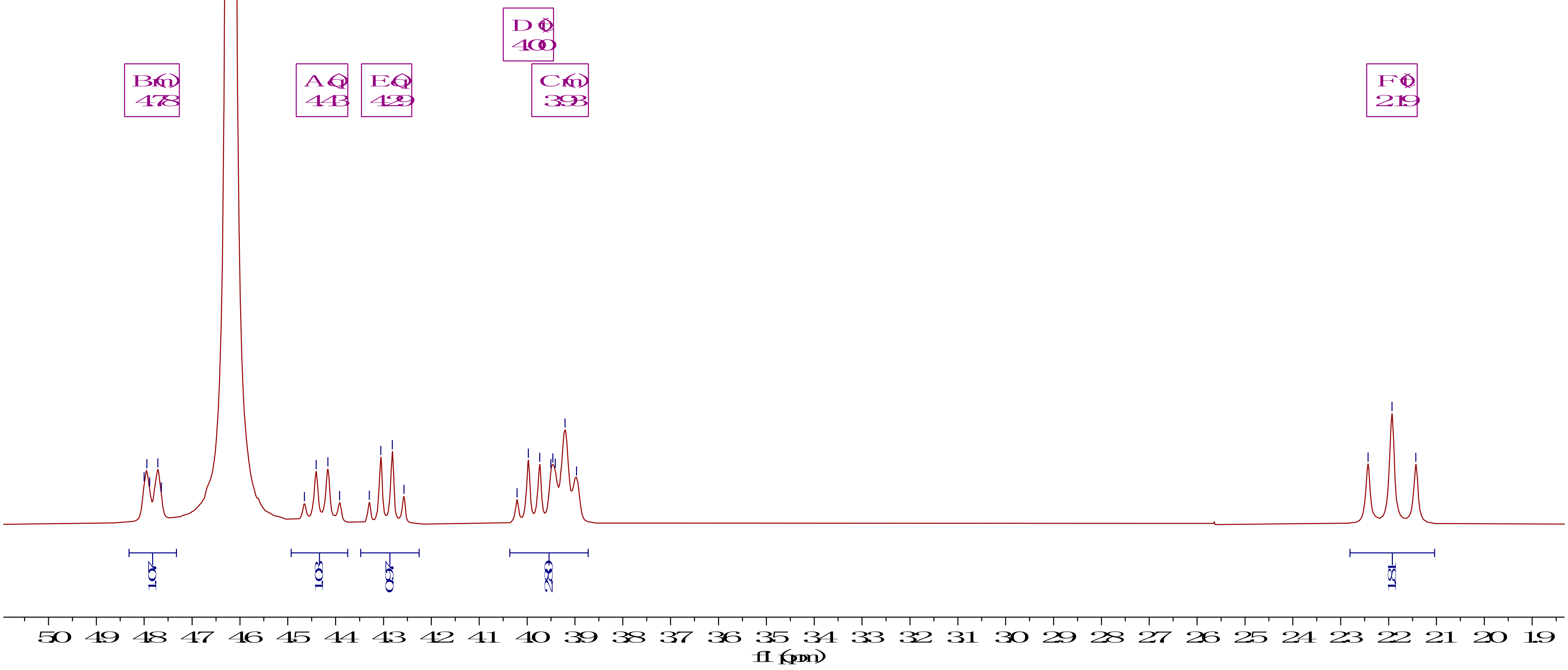

4,6-PCP-InsP$_5$ (**23**), ^{31}P{^{1}H} - NMR (D_2O, 162 MHz)

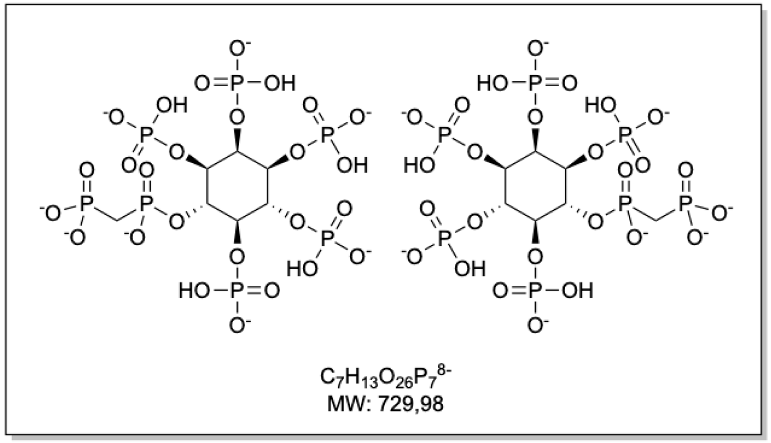

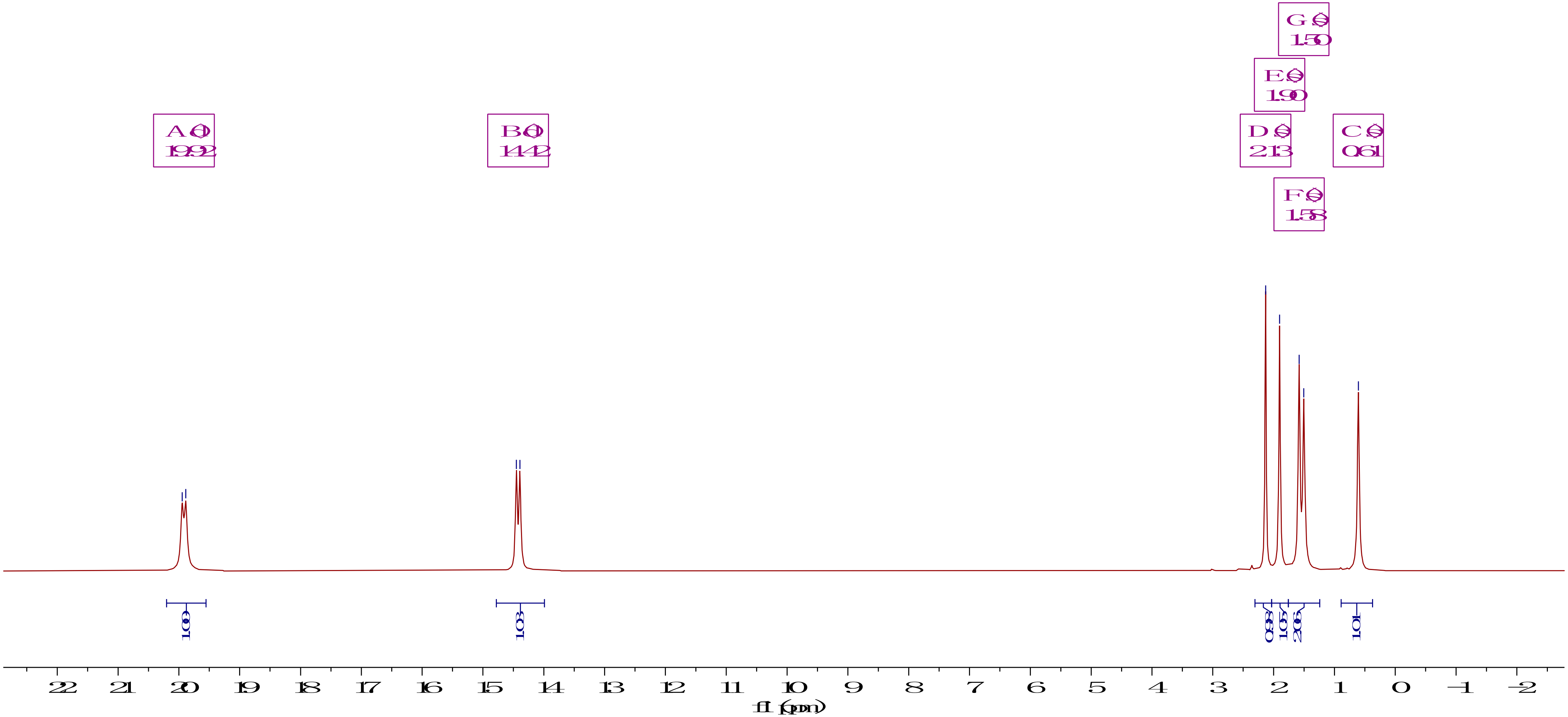

4,6-PCP-InsP$_5$ (**23**), ^{13}C - NMR (D$_2$O, 101 MHz)

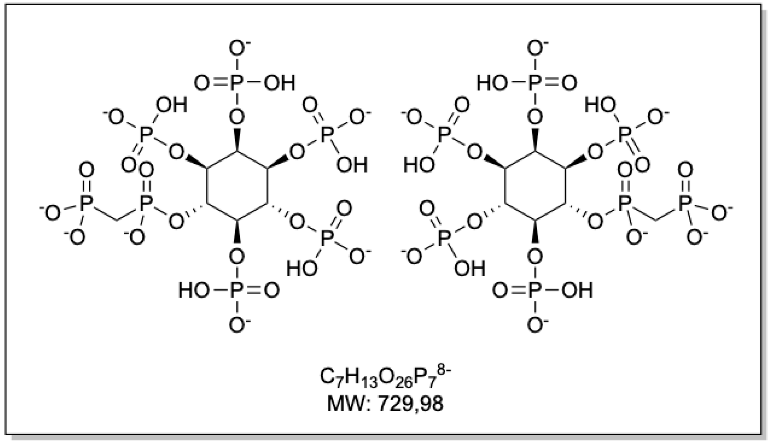

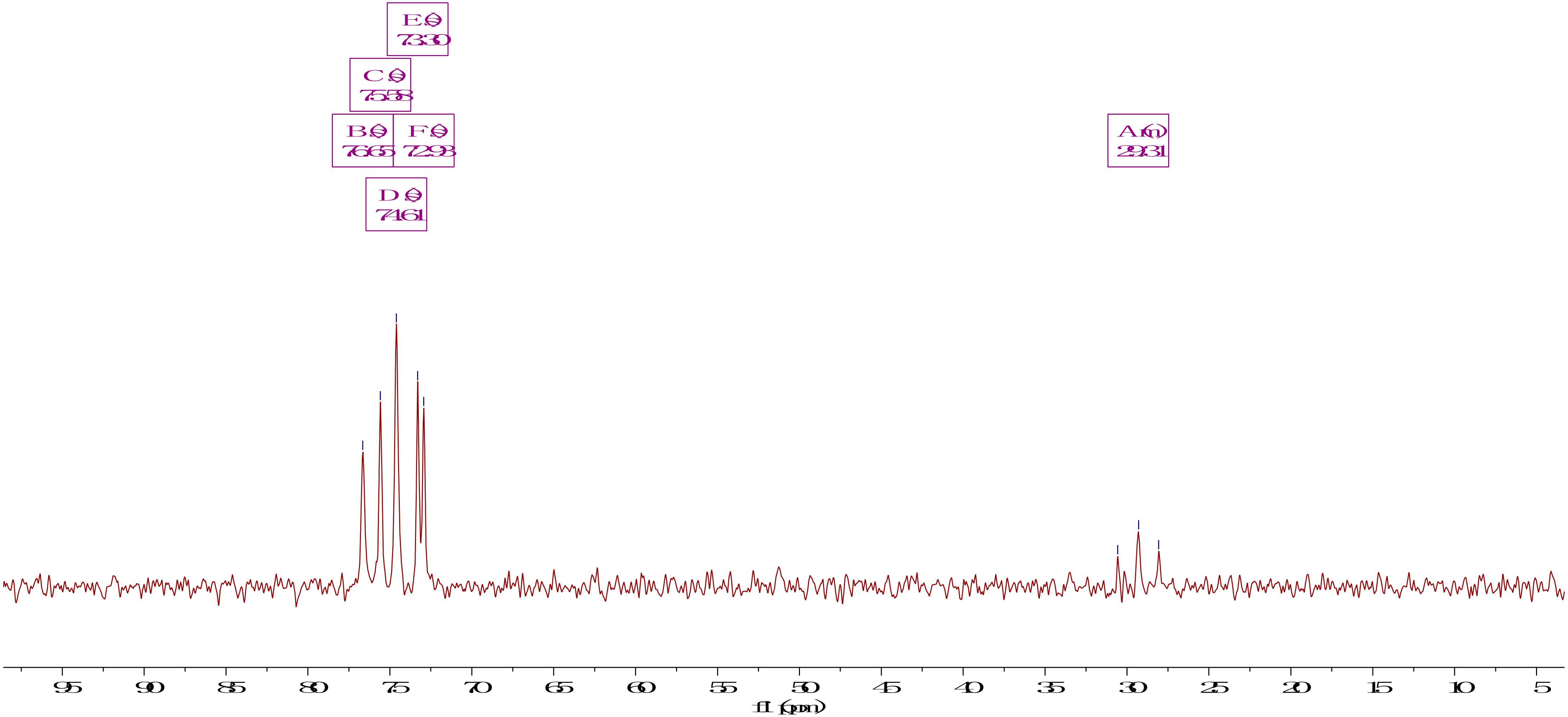

InsP$_2$-derivative **25**, ^{1}H - NMR (MeCN-d_3, 400 MHz)

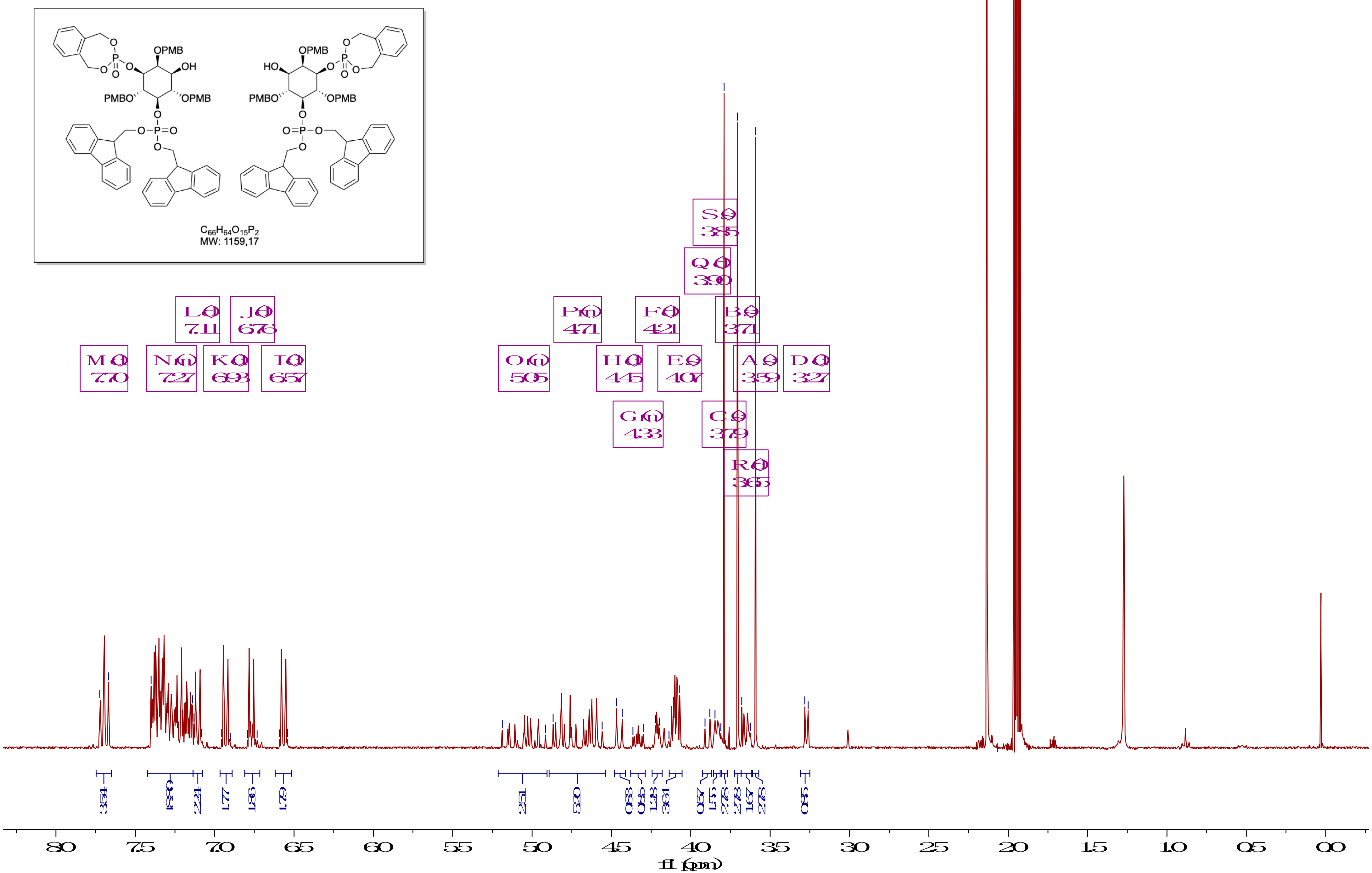

InsP$_2$-derivative **25**, $^{31}P\{^1H\}$ - NMR (MeCN-d_3, 162 MHz)

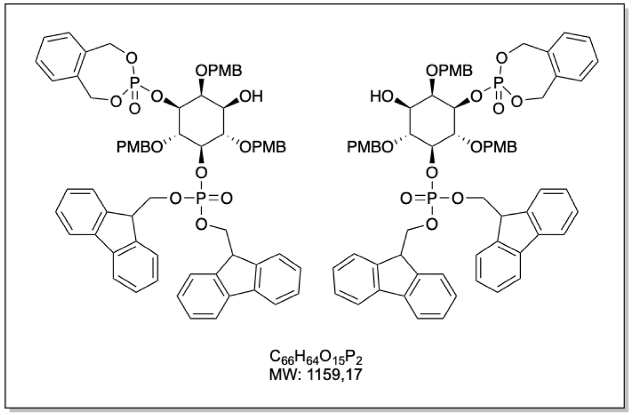

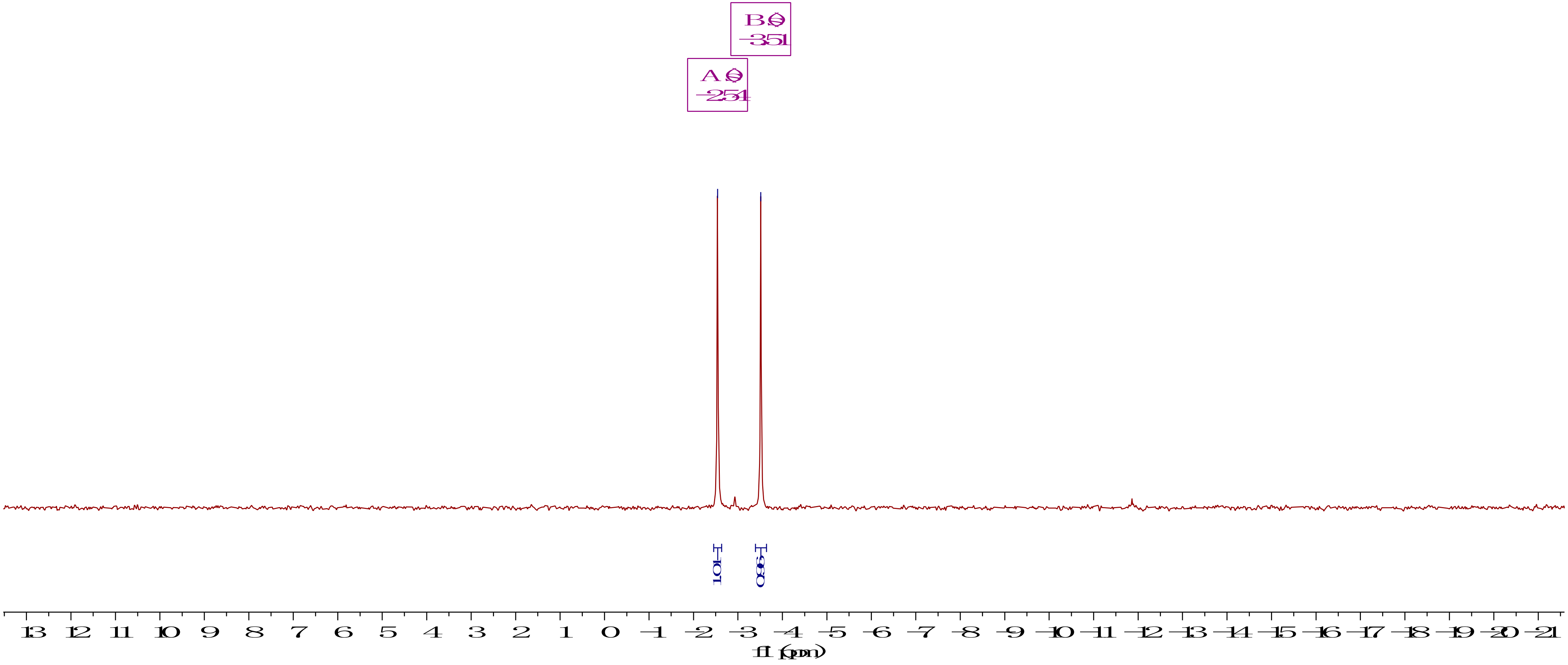

InsP$_2$-derivative **25**, ^{13}C - NMR (MeCN-*d*$_3$, 101 MHz)

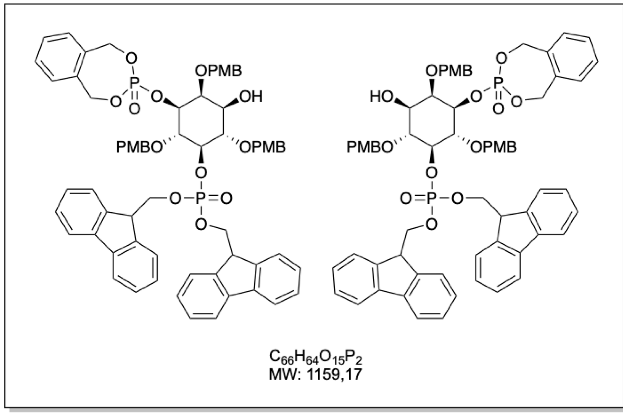

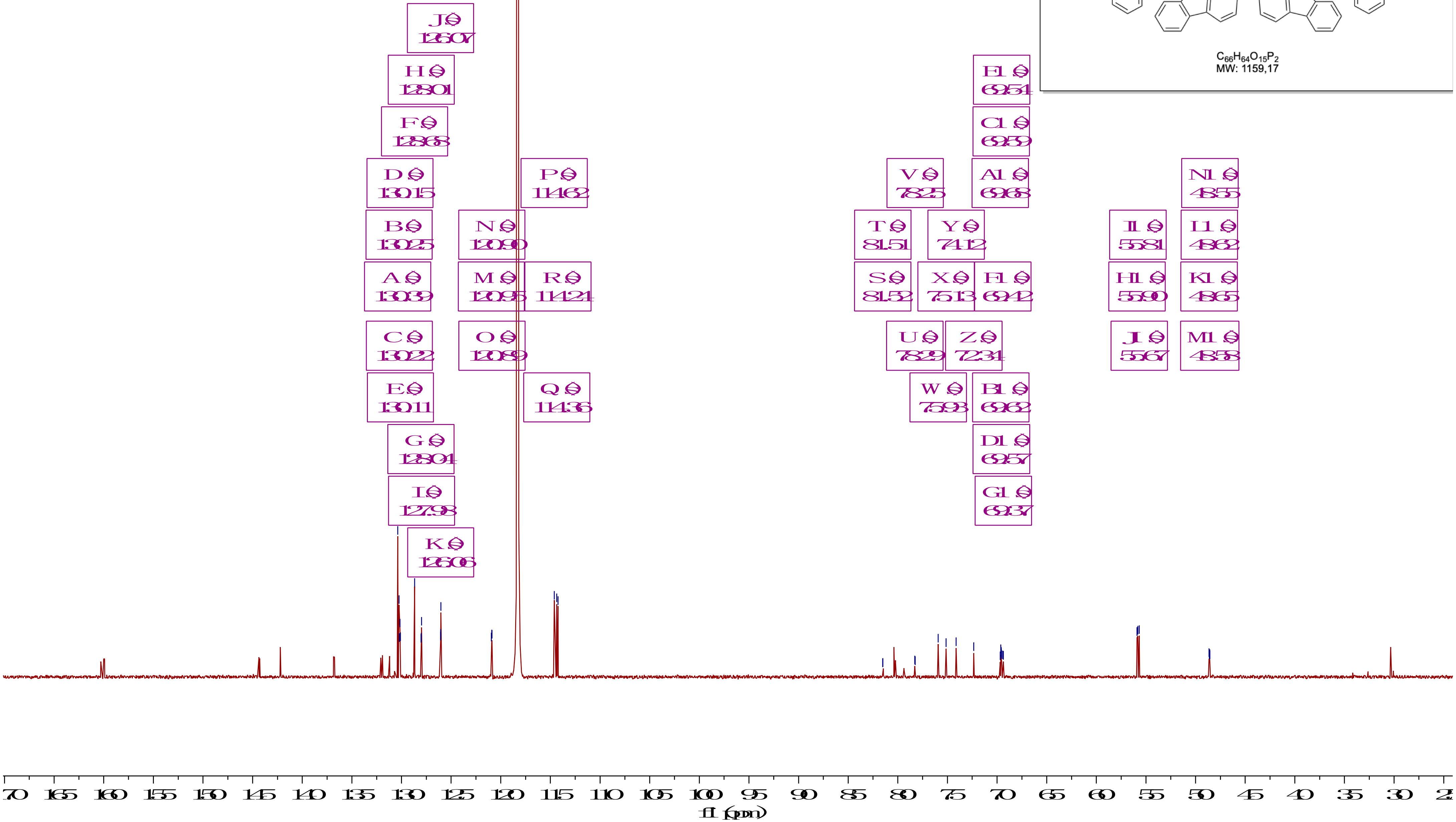

InsP$_2$-derivative **26**, ^{1}H - NMR (MeOH-d_4, 400 MHz)

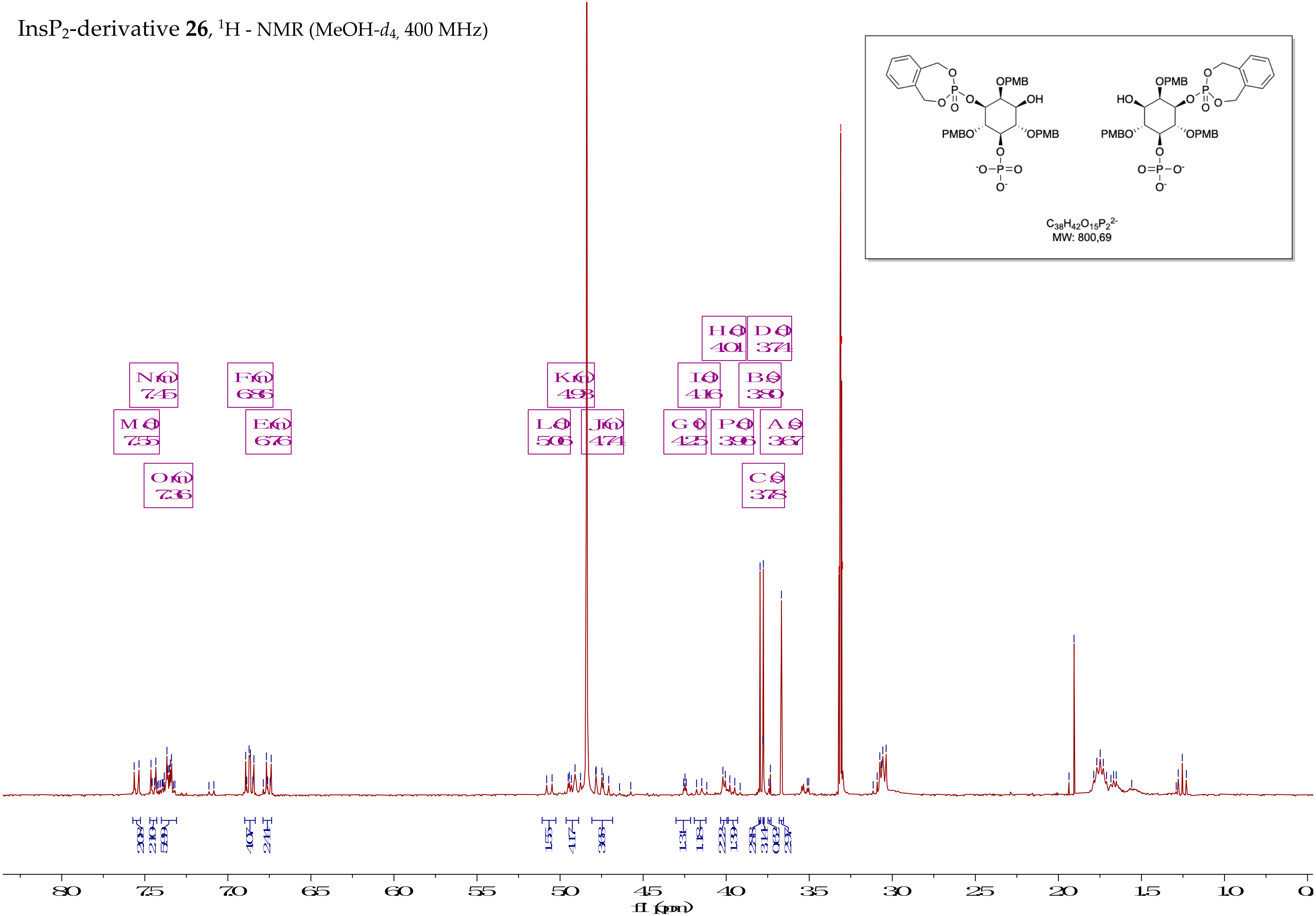

InsP$_2$-derivative **26**, $^{31}P\{^1H\}$ - NMR (MeOH-d_4, 162 MHz)

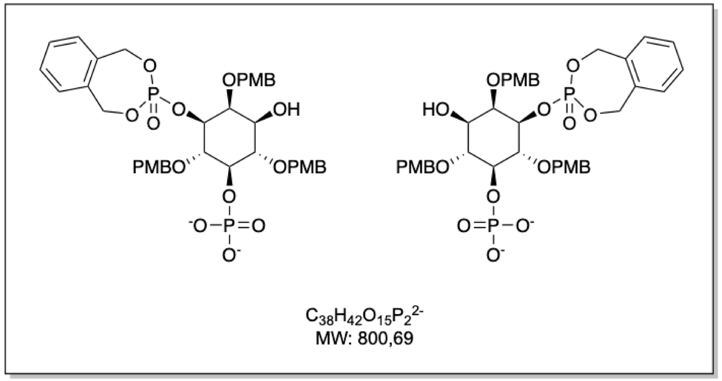

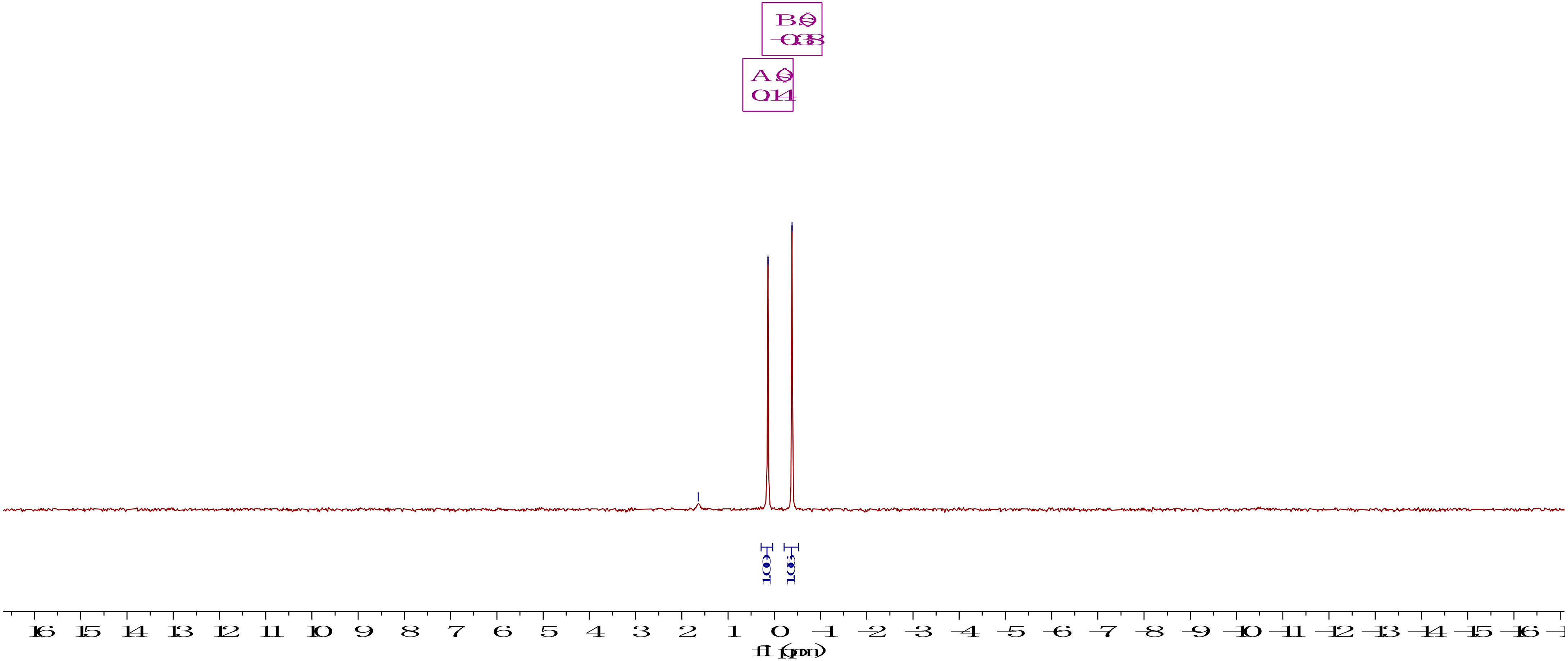

InsP$_2$-derivative **26**, ^{13}C - NMR (MeOH-d_4, 101 MHz)

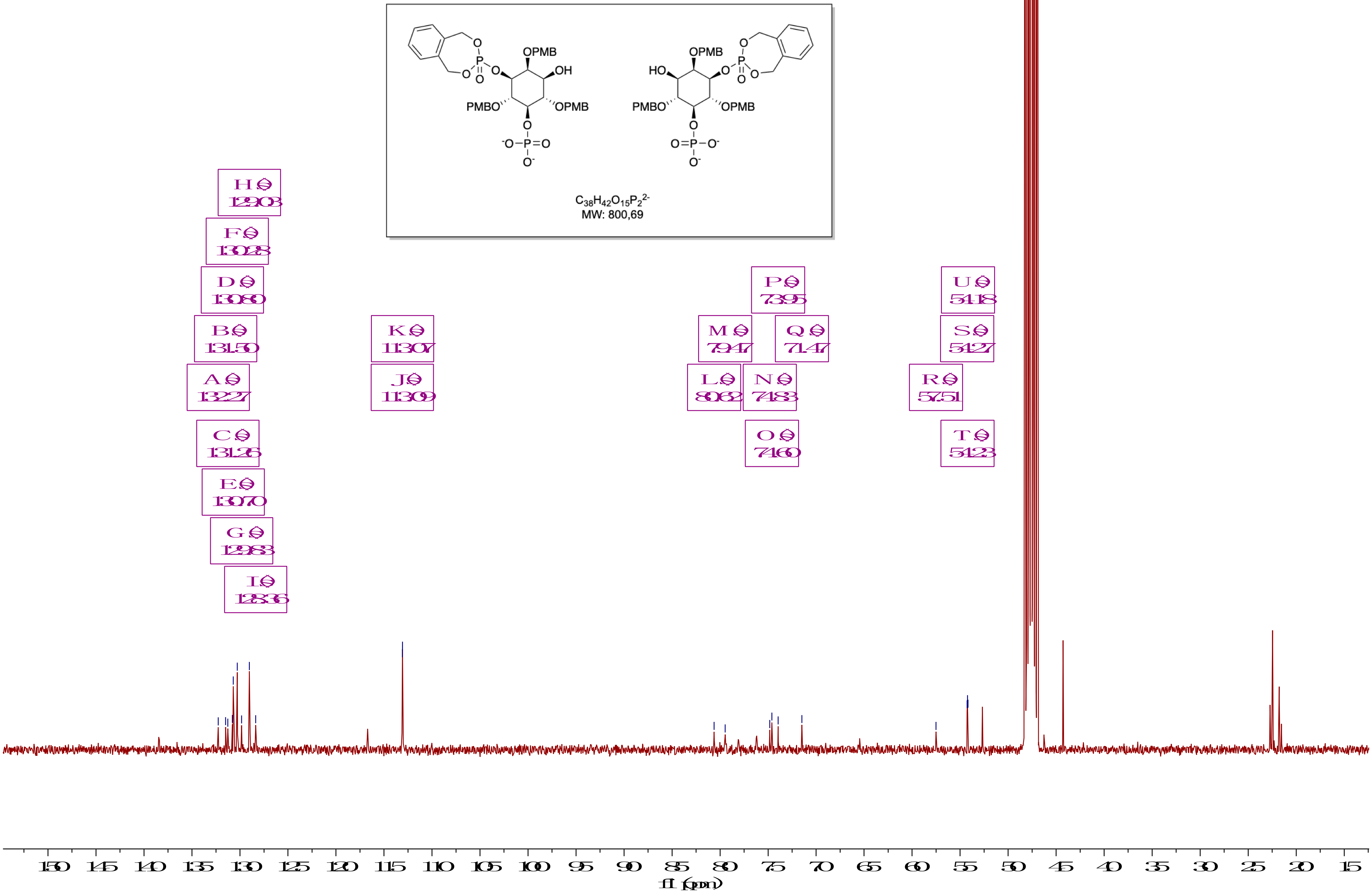

PP-InsP$_1$ derivative **27**, ^{1}H - NMR (MeOH-d_4, 400 MHz)

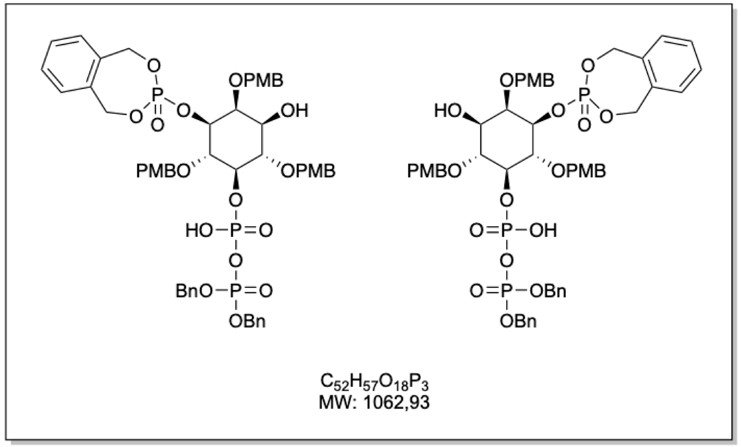

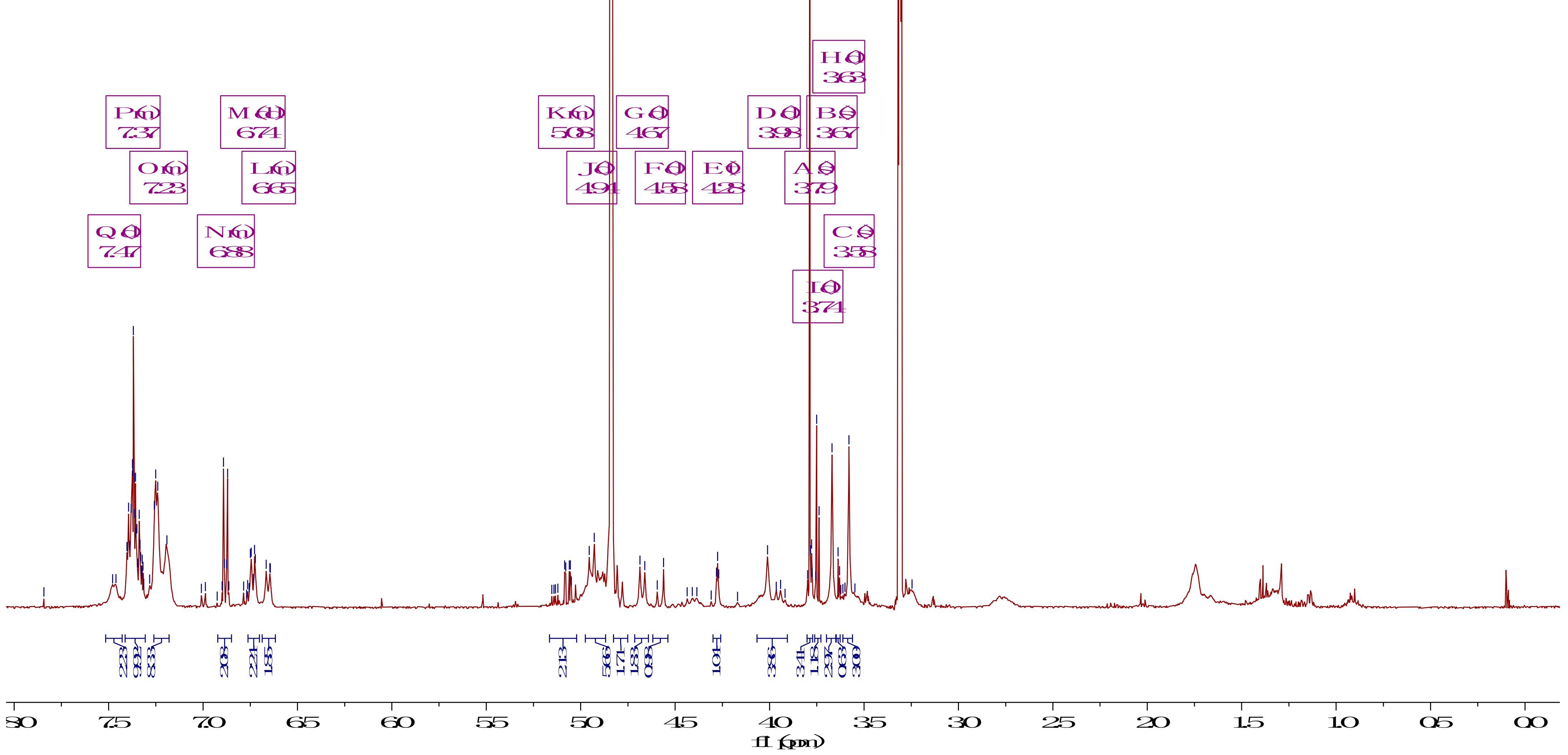

PP-InsP$_1$ derivative **27**, $^{31}P\{^1H\}$ - NMR (MeOH-d_4, 162 MHz)

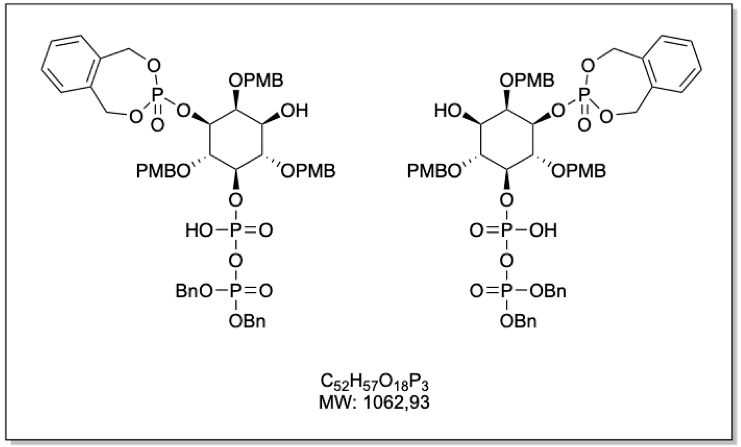

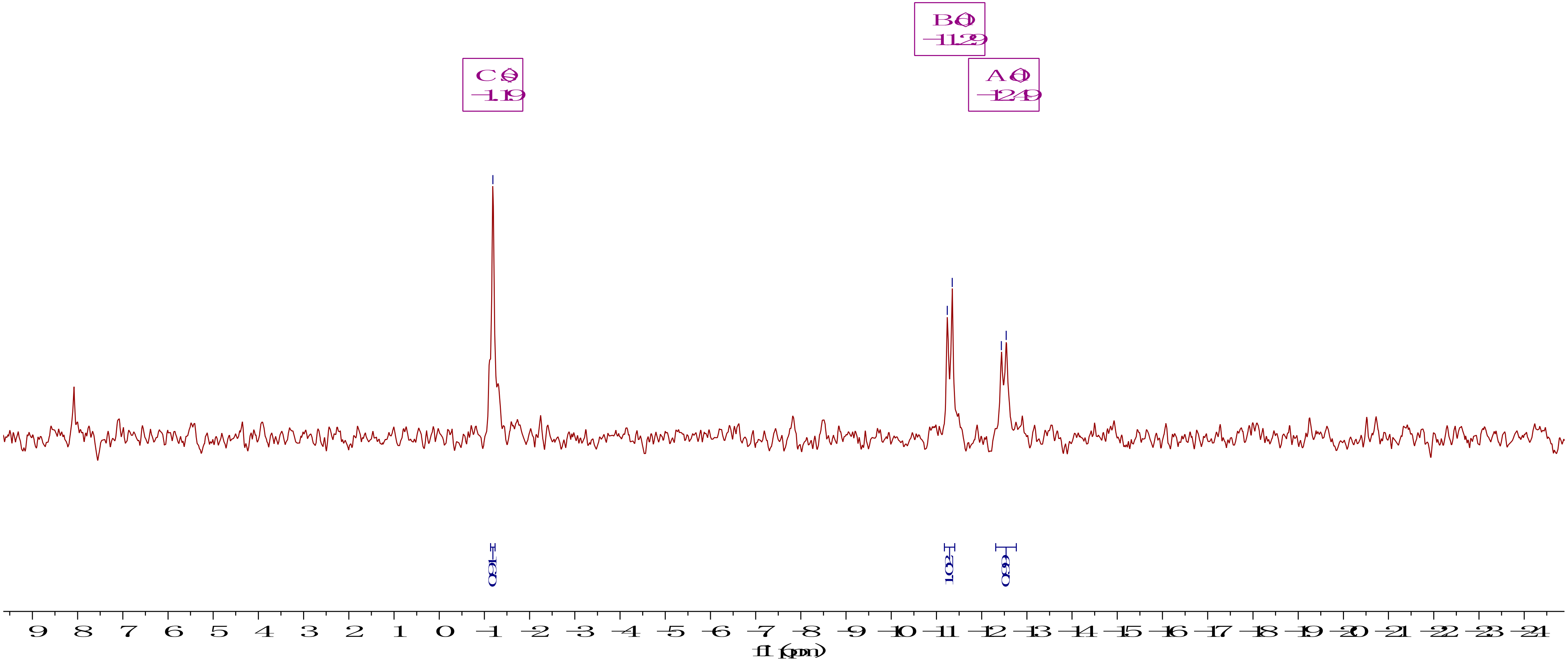

5-PP-1/ 3-InsP$_1$ (**28**), ^{1}H - NMR (D_2O, 400 MHz)

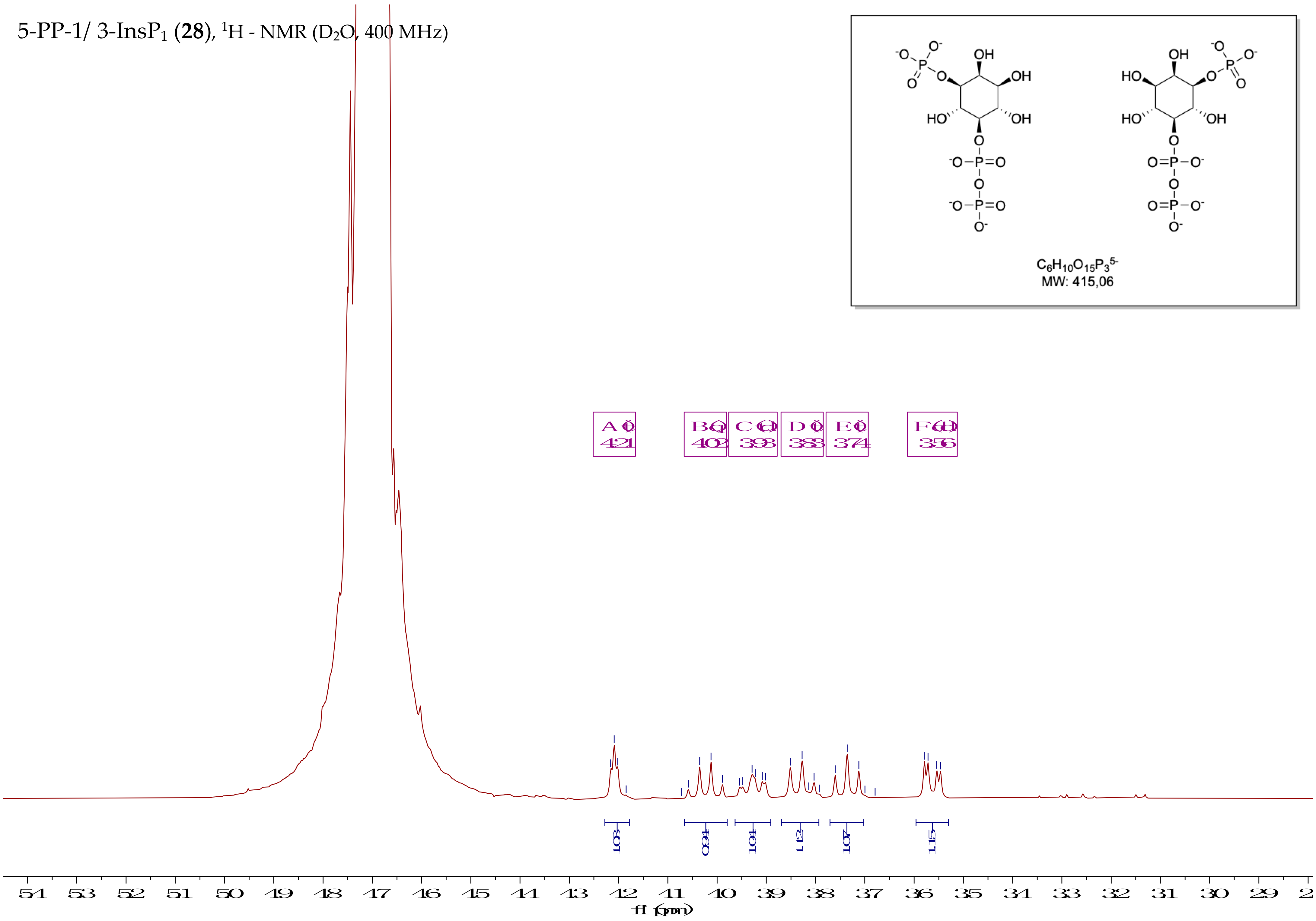

5-PP-1/ 3-InsP$_1$ (**28**), ^{31}P{^{1}H} - NMR (D_2O, 162 MHz)

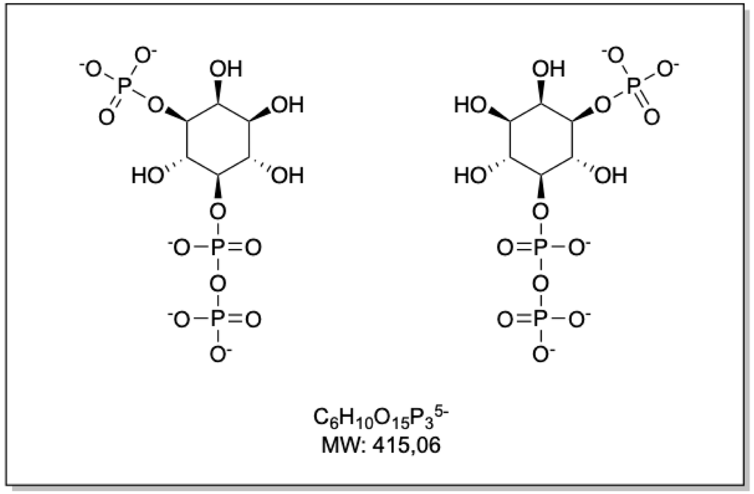

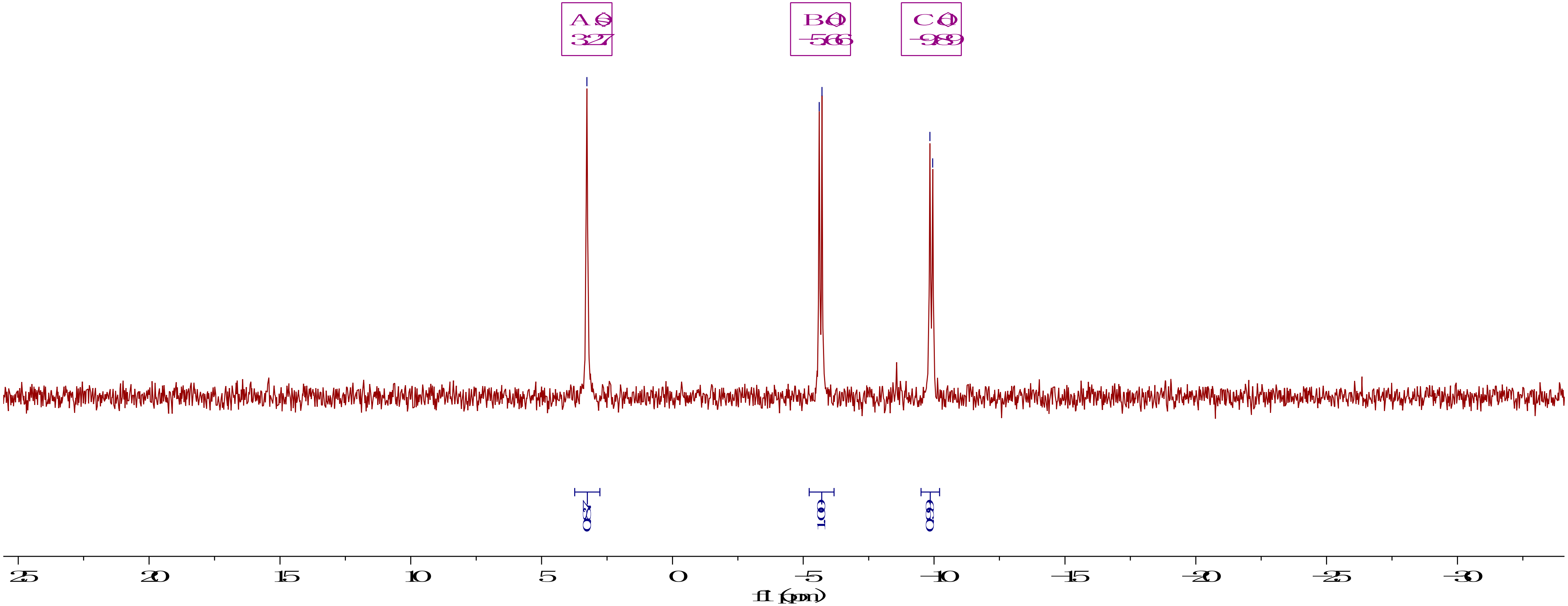

5-PP-1/ 3-InsP$_1$ (**28**), ^{13}C - NMR (D_2O, 101 MHz)

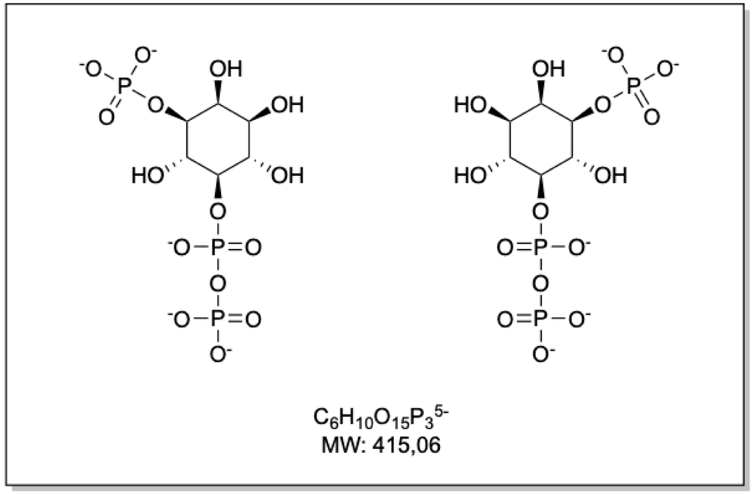

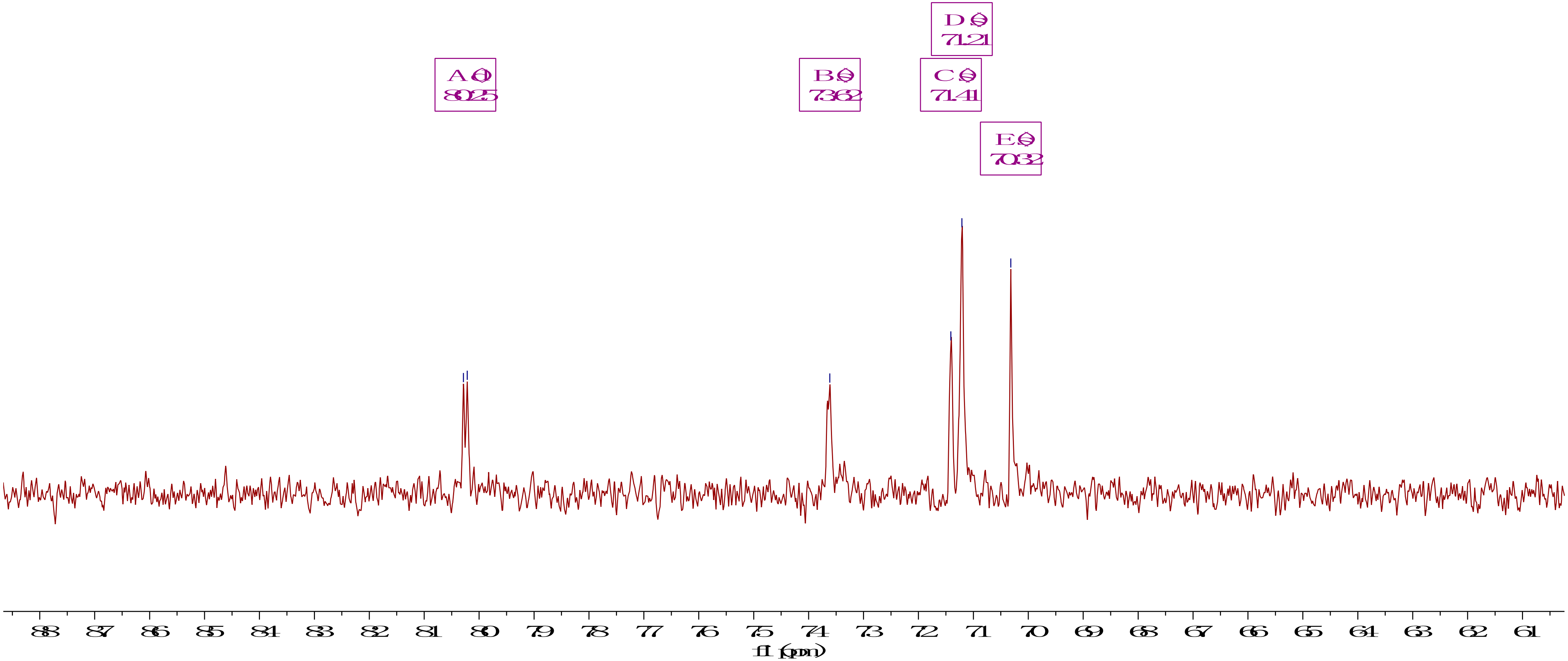

Supplement: Supplementary file 1 [file biomolecules-13-01150-s001.zip › biomolecules-2451466-supplementary.pdf]
